# Global, regional, and national burden of low-back pain attributable to smoking from 1990 to 2021: An age-period-cohort and joinpoint analysis of the Global Burden of Disease Study 2021

**DOI:** 10.18332/tid/226563

**Published:** 2026-09-03

**Authors:** Junkai Kou, Jianhua Ren, Aoying Min, Qi Hao, Shuo Li, Hengrui Chang, Xianzhong Meng

**Affiliations:** 1 Department of Spine Surgery, The Third Hospital of Hebei Medical University, Shijiazhuang, Hebei, China; 2 Department of Geriatric Orthopedics, The Third Hospital of Hebei Medical University, Shijiazhuang, Hebei, China

**Keywords:** low-back pain, smoking, Global Burden of Disease, age-period-cohort analysis, joinpoint analysis

## Abstract

**INTRODUCTION:**

Low-back pain (LBP) is the leading cause of disability worldwide. Although smoking is a well-established, modifiable risk factor that accelerates intervertebral disc degeneration, comprehensive spatiotemporal analyses of its attributable burden remain scarce. This study assessed the global burden of smoking-attributable LBP from 1990 to 2021, elucidating the underlying temporal dynamics and socioeconomic inequalities.

**METHODS:**

Data were extracted from the Global Burden of Disease (GBD) study 2021. We evaluated absolute disability-adjusted life years (DALYs), age-standardized DALYs rate (ASDR), and estimated annual percentage change (EAPC) across 204 countries and territories. To disentangle temporal trends, age-period-cohort and joinpoint regression models were applied. The spatial-temporal relationship between the burden and the sociodemographic index (SDI) was also examined.

**RESULTS:**

Globally, the absolute number of DALYs for smoking-attributable LBP increased by 29.6% to 8.67 million in 2021, primarily driven by population growth and aging. Conversely, the global ASDR decreased significantly by 35.8% to 99.95. Males bore a substantially higher burden than females, with biological risk peaking at 60–69 years (inverted U-shape). The ASDR trajectory exhibited a distinct inverted U-shaped relationship with SDI; while high-income nations experienced rapid declines (e.g. Denmark, EAPC= -2.29), the burden surged in specific developing regions (e.g. Afghanistan, EAPC=2.37). Joinpoint analysis revealed an accelerated global decline from 2016 to 2021 (annual percent change, APC= -1.70). Age-period-cohort modeling confirmed continuous monotonic reductions in both period and cohort relative risks, highlighting a profound ‘generational dividend’.

**CONCLUSIONS:**

Despite continuous improvements in age-standardized rates driven by recent global tobacco control policies and younger birth cohorts, the absolute burden of smoking-attributable LBP continues to grow. Progress is alarmingly unequal, heavily favoring high-income countries while intensifying in developing nations. Global health strategies must prioritize equitable implementation of tobacco control in lower income regions and integrate cessation programs into occupational health initiatives and strengthening of targeted tobacco control policies, particularly in low- and middle-income regions.

## INTRODUCTION

Low-back pain (LBP) represents a massive global health challenge and remains the leading cause of years lived with disability (YLD) worldwide across all age groups^1^. Beyond the profound physical suffering and diminished quality of life experienced by individuals, LBP imposes a socioeconomic burden on healthcare systems through direct medical costs and indirect productivity losses, such as absenteeism and early retirement^2,3^. As the global population undergoes rapid aging and structural demographic shifts, the absolute number of people living with LBP is projected to increase substantially, making it a pivotal priority for global public health interventions^4^.

While the etiology of LBP is notoriously complex and multifactorial – encompassing occupational hazards, obesity, and ergonomic factors – smoking has been consistently identified as a crucial, yet entirely preventable, risk factor^5^. Epidemiological evidence suggests a strong dose-response relationship between current smoking and the prevalence, incidence, and severity of LBP^6^. Biologically, the structural integrity of the intervertebral discs relies on a delicate microvascular supply. Nicotine and carbon monoxide from tobacco smoke induce potent vasoconstriction and endothelial dysfunction, leading to accelerated disc degeneration and impaired healing^7^. Furthermore, smoking may alter central pain processing and amplify pain perception, while chronic smoker’s cough increases mechanical stress and intra-abdominal pressure on the lumbar spine^8^. Consequently, targeted tobacco control could serve as a highly effective upstream strategy to mitigate the global burden of LBP.

Although previous iterations of the Global Burden of Disease (GBD) study have documented the general epidemiology of LBP^9^, critical knowledge gaps remain regarding the specific burden attributable to smoking, particularly in light of recent global events and evolving tobacco control policies. Most existing literature relies on older datasets (e.g. GBD 2019) that do not capture the disruptions of the COVID-19 pandemic era or the latest demographic transitions^10^. More importantly, traditional descriptive analyses of age-standardized rates fail to disentangle the complex underlying temporal dynamics. To date, there is a distinct lack of comprehensive research utilizing advanced statistical modeling, such as joinpoint regression, to identify precise structural shifts (inflection points) in disease trends over the past three decades. Furthermore, an age-period-cohort framework is urgently needed to separate the intrinsic physiological effects of aging from historical period influences (e.g. healthcare advancements or global crises) and birth cohort effects (e.g. generational shifts in smoking prevalence)^11^.

To address these gaps, this study utilized the most recent data from the GBD Study 2021 to provide a systematic and updated assessment of the spatiotemporal trends in the burden of LBP attributable to smoking across 204 countries and territories from 1990 to 2021. By applying joinpoint regression and an age-period-cohort modeling framework, alongside an analysis across different levels of the sociodemographic index (SDI), this study aims to describe the temporal trends and socioeconomic variations associated with this burden. We expect that these findings may provide observational evidence to help evaluate the broader impact of tobacco control measures and inform the development of age- and cohort-appropriate public health strategies.

## METHODS

### Data source

The present study used publicly accessible estimates from the Global Burden of Disease (GBD) Study 2021. Data describing the burden of LBP attributable to smoking were obtained through the Global Health Data Exchange (GHDx) results tool. Estimates were available for 204 countries and territories and were subsequently grouped into 21 GBD regions and five SDI quintiles.

We extracted the annual absolute numbers and rates of disability-adjusted life years (DALYs) by sex (both, male, female), year (1990 to 2021), and 5-year age groups (from 30–34 years up to ≥95 years). DALYs are a standard metric in GBD, calculated as the sum of years of life lost (YLL) due to premature mortality and years lived with disability (YLD). Since LBP is generally not considered a direct cause of death in the GBD framework, the DALYs for LBP are entirely equivalent to YLD. Ethical approval and informed consent were waived for this study because the GBD database contains anonymized, aggregated, and publicly available macro-level data. This study was conducted in compliance with the Guidelines for Accurate and Transparent Health Estimates Reporting (GATHER)^12^.

### Case definition and risk factors

In the GBD 2021 framework, LBP was defined as pain localized to the posterior aspect of the spine (from the lower margin of the twelfth rib to the lower gluteal folds) lasting for at least one day, mapped to the International Classification of Diseases, Tenth Revision (ICD-10) codes M54.3 (Sciatica), M54.4 (Lumbago with sciatica), M54.5 (Low-backpain), M54.8 (Other dorsalgia), and M54.9 (Dorsalgia, unspecified)^1^.

In this study, ‘smoking’ encompasses the use of any smoked tobacco product. The GBD 2021 framework classifies exposure into two primary categories: current smokers (defined as individuals who currently use any smoked tobacco product on a daily or occasional basis) and former smokers (individuals who have quit using all smoked tobacco products for at least six months). Cumulative smoking exposure was additionally characterized using continuous measures, including cigarettes smoked per smoker per day and pack-years. These exposure distributions were estimated from nationally representative household survey data using spatiotemporal Gaussian process regression (ST-GPR).

Smoking-attributable LBP burden was estimated using the population attributable fraction (PAF). The PAF reflects the proportion of burden that could theoretically be avoided if population exposure were reduced to the theoretical minimum risk exposure level (TMREL), defined as zero exposure or never smoking. Briefly, for a continuous risk factor like smoking exposure x, the PAF for a given age, sex, location, and year was calculated as:


$$\text{PAF}=\frac{\int_{\text{x=0}}^{\text{m}}\text{RR}\left(\text{x}\right)\text{P}(\text{x})\text{dx}-\text{RR}\left(\text{TMREL}\right)}{\int_{\text{x=0}}^{\text{m}}\text{RR}\left(\text{x}\right)\text{ P}(\text{x})\text{dx}}$$

where RR(x) represents the age-specific relative risk at exposure level x, P(x) denotes the population distribution of exposure, m is the maximum exposure level, and RR (TMREL)=1. Further details of the GBD approach to PAF estimation, including comparisons between observed exposure distributions and the counterfactual distribution at the TMREL, are available elsewhere^13^. In GBD 2021, relative risks were estimated using the Meta-Regression-Bayesian, Regularized, Trimmed (MR-BRT) framework. For musculoskeletal outcomes such as LBP, age-specific dose-response functions were incorporated to account for variation in excess smoking-related risk across age groups^14^.

### Burden metrics and sociodemographic index

Age-standardized rates were calculated by direct standardization to the GBD world standard population, thereby improving comparability across locations and time periods with different population age structures^15^. Socioeconomic development was assessed using the SDI, which integrates lag-distributed income per capita, education level among individuals aged ≥15 years, and fertility among those aged <25 years^14,16^. Countries and territories were classified into low, low-middle, middle, high-middle, and high SDI groups. For age-related analyses, the original 5-year age categories were further combined into five broader groups: 30–49, 50–59, 60–69, 70–79, and ≥80 years^13^.

### Statistical analysis

*Descriptive analysis and trend estimation*

To analyze the temporal trends in the burden of LBP attributable to smoking from 1990 to 2021, we calculated the percentage change in the absolute number of DALYs. The estimated annual percentage change (EAPC) was employed to quantify the temporal trends in ASR. The EAPC was calculated using a generalized linear regression model fitted to the natural logarithm of the ASR, following the formula:

ln (ASR) = α+βx+ϵ, where x represents the calendar year, and ϵ is the error term. The EAPC and its 95% confidence interval (CI) were derived as: EAPC=100×[exp(β)-1]. Temporal trends were classified according to the 95% CI of the EAPC. An increase was considered statistically significant when the entire 95% CI was above zero, whereas a significant decrease was defined when the entire interval was below zero.

*Joinpoint regression analysis*

To identify specific time points where the trends in ASRs significantly shifted, we performed joinpoint regression analysis using the Joinpoint Regression Program (Version 5.1.0.0, Statistical Research and Applications Branch, National Cancer Institute). This method identifies the optimal number of inflection points (joinpoints) using a grid search method and permutation tests. For each identified segment, we calculated the annual percentage change (APC) to describe the slope of the trend. To summarize the trend over the entire study period (1990–2021), the average annual percentage change (AAPC) was computed as a weighted average of the APCs from the joinpoint model, with weights equal to the length of each segment.

*Age-period-cohort analysis*

To disentangle the independent effects of chronological age, time period, and birth cohort on the disease burden, we constructed an age-period-cohort model. The input data were arranged into consecutive 5-year age groups (ranging from 30–34 to ≥95 years) and 5-year time periods (from 1990–1994 through 2015–2019, with 2020–2021 data adjusted to align with model constraints). This analysis focused on estimating several key parameters: Net Drift, which represents the overall annual percentage change in the expected age-adjusted rates over time (analogous to the EAPC but adjusted for age and cohort distributions); Local Drift, which captures the annual percentage change in rates for each specific age group; and the Longitudinal Age Curve, which reflects the fitted age-specific rates in the reference cohort adjusted for period effects to represent the biological risk associated with aging. Additionally, the model estimated the Period Effect (expressed as relative risk, RR), reflecting risk variations over time influenced by immediate factors affecting all age groups simultaneously (e.g. changes in diagnostic criteria or treatments), and the Cohort Effect (expressed as RR), which highlights risk variations across birth cohorts representing early-life exposures and generational lifestyle differences. Both period and cohort effects were referenced to their respective medians. To address the inherent collinearity problem (age = period - cohort), we utilized the intrinsic estimator method to ensure parameter identifiability^17^.

*Statistical software and significance*

All statistical analyses were performed using R software (Version 4.3.1, R Foundation for Statistical Computing). The age-period-cohort analysis was conducted using the Epi package or comparable tools within the R environment. A p<0.05 was considered statistically significant. All statistical tests were two-sided. The 95% uncertainty intervals (UIs) provided by the GBD study were reported for absolute numbers and rates, while 95% CIs were calculated for trend estimates (EAPC, APC, and AAPC).

## RESULTS

### Global burden and temporal trends

In 2021, the global absolute number of DALYs for LBP attributable to smoking reached 8.67 million (95% UI: 4.55–13.39), representing a significant 29.60% increase since 1990. However, after accounting for population growth and aging, the global ASDR decreased substantially by 35.83%, dropping to 99.95 (95% UI: 52.62–154.06) in 2021. The overall net drift indicated a continuous annual decline of -0.90% (95% CI: -0.96 – -0.85) over the 32-year period (Table 1).

**Research PaperTobacco Induced Diseases Research PaperTobacco Induced Diseases Table 1:** Global trends in DALYs of low-back pain attributable to smoking by sex, SDI, and WHO regions, 1990–2021

	*DALYs*		*All-age DALYs rate per 100000*		*ASDR*		*Net drift of DALYs % per year (95% CI)*
	*2021 n (95% UI)*	*1990–2021 Percent change (95% UI)*	*2021 Rate (95% UI)*	*1990–2021 Percent change (95% UI)*	*2021 Rate (95% UI)*	*1990–2021 Percent change (95% UI)*	
**Global**	8667593.77 (4546840.97–13394437.13)	29.6 (12.57–44.91)	109.4 (57.39–169.06)	-14.26 (-25.53 – -4.13)	99.95 (52.62–154.06)	-35.83 (-44.27 – -28.05)	-0.9 (-0.96 – -0.85)
**Sex**							
Male	5826090.88 (3117606.51–8726541.1)	34.21 (21.89–46.44)	146.48 (78.38–219.4)	-10.93 (-19.1 – -2.81)	136.92 (73.22–205.29)	-33.47 (-39.44 – -27.82)	-0.88 (-0.94 – -0.81)
Female	2841502.89 (1444755.77–4534672.56)	21.06 (-4.03–47.02)	72.02 (36.62–114.94)	-20.17 (-36.71 – -3.05)	64.29 (32.93–102.5)	-40.51 (-53.16 – -27.46)	-1.08 (-1.19 – -0.97)
**SDI**							
High	5317183.97 (2733961.84–8135004.46)	17.93 (4.78–32.18)	210.15 (108.05–321.51)	-4.51 (-15.16–7.03)	150.64 (78.59–226.63)	-28.58 (-36.31 – -21.41)	-0.41 (-0.48 – -0.33)
High-middle	1343222.99 (730853.41–2066996.8)	35.1 (13.96–58.02)	83.02 (45.17–127.76)	-5.81 (-20.55–10.17)	68.66 (37.44–105.84)	-42.37 (-51.46 – -32.61)	-0.21 (-0.33 – -0.08)
Middle	790012.05 (429271.77–1201721.69)	92.22 (60.62–125.93)	83.54 (45.39–127.07)	21.34 (1.39–42.62)	82.47 (44.63–125.97)	-21.49 (-34.26 – -7.97)	-0.83 (-0.92 – -0.74)
Low-middle	548923.05 (287635.15–904682.19)	34.99 (5.03–68.99)	47.27 (24.77–77.9)	-18.04 (-36.23–2.6)	54.35 (28.62–89.77)	-39.08 (-52.93 – -23.59)	-1.19 (-1.34 – -1.03)
Low	656315.59 (354688.53–1055224.18)	77.83 (34.27–133.05)	39.5 (21.35–63.51)	-17.49 (-37.7–8.12)	61.56 (33.03–99.27)	-27.63 (-45.2 – -4.97)	-0.7 (-0.81 – -0.58)
**WHO regions**							
African Region	330258.48 (174635.3–545745.1)	99.83 (32.51–190.78)	28.21 (14.92–46.62)	-17.14 (-45.06–20.57)	46.83 (24.86–77.12)	-25.82 (-51.22–9.12)	-1.1 (-1.16 – -1.04)
Eastern Mediterranean Region	565154.07 (305654.91– 909912.58)	155.81 (72.93–261.43)	75.84 (41.02–122.1)	24.84 (-15.61–76.38)	92.82 (50.57–148.8)	-11.02 (-40.32–25.48)	-0.2 (-0.29 – -0.1)
European Region	2574154.31 (1330505.95–3959833.01)	12.7 (-2.84–26.91)	272.39 (140.79–419.03)	2.46 (-11.67–15.38)	199.51 (105.38–302.15)	-15.59 (-26.42 – -5.35)	-0.3 (-0.41 – -0.19)
Region of the Americas	1557592.83 (770787.88–2398251.31)	17.66 (3.03–33.82)	152.65 (75.54–235.04)	-18.09 (-28.28 – -6.84)	127.45 (62.85–195.28)	-40.05 (-47.51 – -31.55)	-0.99 (-1.1 – -0.89)
South-East Asia Region	832260.07 (429234.82–1303437.9)	28.5 (2.69–61.95)	46.48 (23.97–72.8)	-21.7 (-37.43 – -1.32)	48.73 (25.39–76.68)	-45.76 (-56.62 – -31.78)	-1.41 (-1.56 – -1.25)
Western Pacific Region	2746392.44 (1495103.01–4127621.16)	35.99 (16.78–56.9)	124.13 (67.57–186.56)	5.58 (-9.33–21.82)	92.52 (50.86–139.92)	-34.47 (-43.05 – -25.23)	-0.9 (-1.04 – -0.76)

ASDR: age-standardized DALYs rate per 100000 population. The all-age DALYs rate is equivalent to the crude DALYs rate. Net drift represents the estimated overall annual percentage change in DALYs, adjusting for period and cohort effects. DALYs: disabilityadjusted life years. SDI: sociodemographic index. UI: uncertainty interval. CI: confidence interval.

Pronounced sex disparities were observed. In 2021, males bore more than twice the ASDR (136.92) compared to females (64.29). Despite this higher baseline burden in males, both sexes experienced significant downward trends in their ASDRs from 1990 to 2021, with females demonstrating a slightly faster annual reduction (net drift = -1.08%) than males (net drift = -0.88%).

The burden of LBP attributable to smoking varied considerably across socioeconomic development levels. In 2021, the high SDI region recorded the highest ASDR (150.64), while the low-middle and low SDI regions exhibited much lower rates (54.35 and 61.56, respectively). Although all five SDI quintiles achieved reductions in their ASDRs between 1990 and 2021, the low-middle SDI region experienced the fastest annual decline (net drift = -1.19%), whereas the high-middle SDI region showed the slowest progress (net drift = -0.21%) (Table 1).

At the regional level, the European Region exhibited the highest ASDR globally in 2021 (199.51), followed by the Region of the Americas (127.45). Conversely, the African and South-East Asia regions reported the lowest ASDRs. Over the 32-year study period, the absolute DALYs surged dramatically in the Eastern Mediterranean (155.81%) and African (99.83%) regions due to demographic expansions. Nevertheless, all six WHO regions successfully reduced their ASDRs, with the South-East Asia Region demonstrating the most substantial annual improvement (net drift = -1.41%) and the European Region showing the least (net drift = -0.30%).

### Age-specific trends and demographic shifts

**Figure 1:**
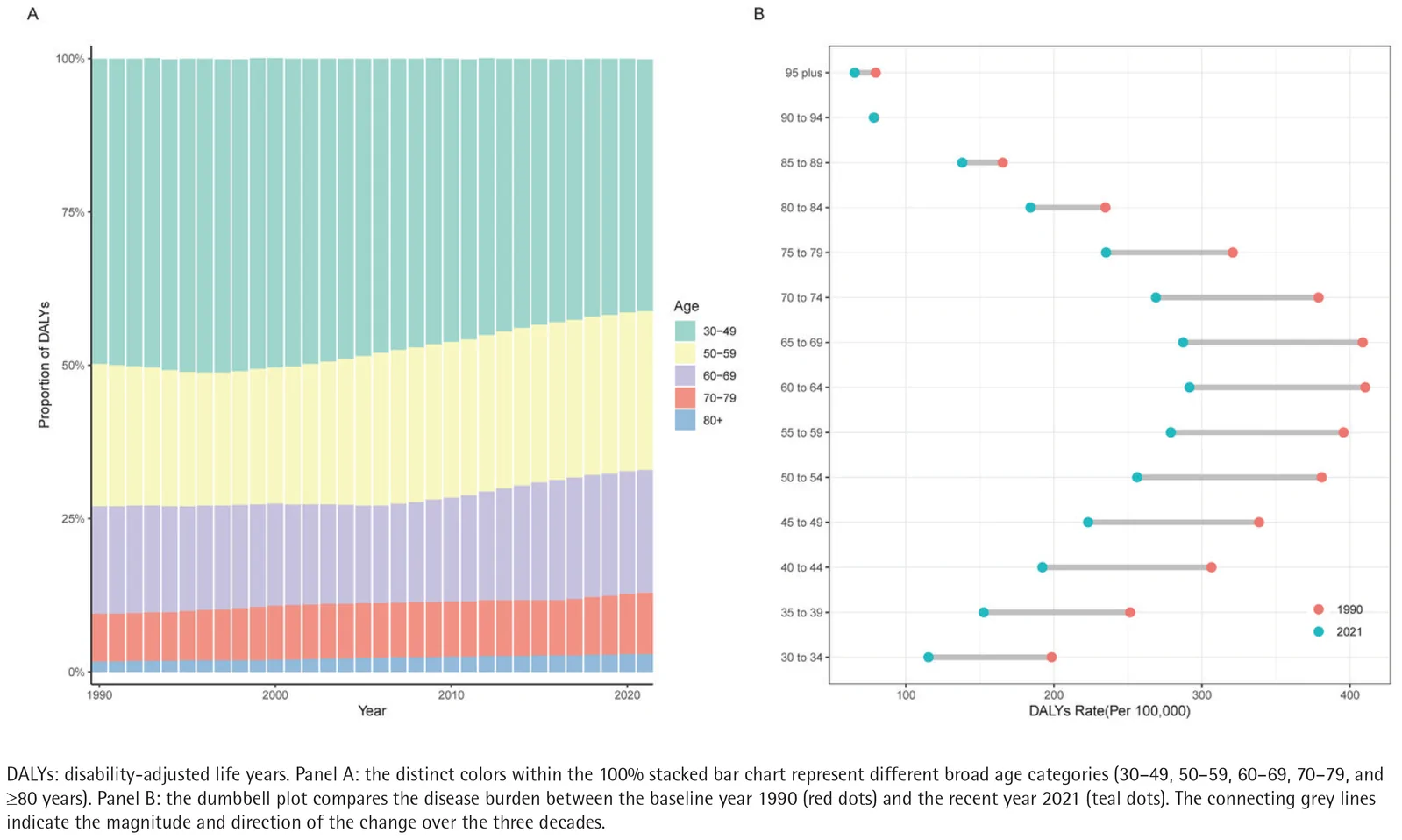
Global trends in the proportion and age-specific DALYs rate of low-back pain caused by smoking, 1990–2021: A) Temporal trends in the age composition (proportion) of total DALYs for both sexes combined globally; B) Changes in age-specific DALYs rate across detailed 5-year age groups globally

Analysis of the age composition revealed a distinct demographic shift in the burden of LBP attributable to smoking over the past three decades. As illustrated in Figure 1A, the proportion of total DALYs accounted for by younger adults (aged 30–49 years) progressively shrank from 1990 to 2021. Conversely, the burden proportion among older age demographics – particularly those aged 60–69, 70–79, and ≥80 years – expanded substantially over time. This aging pattern of the disease burden was observed to be highly consistent across both males and females (Supplementary file Figures 1A and 1C).

Despite the growing proportion of the burden concentrated in older populations, the actual age-specific DALYs rate uniformly declined across all 5-year age groups from 1990 to 2021 (Figure 1B). The distribution of age-specific rates displayed a characteristic inverted U-shape across the lifespan. The burden escalated with age, peaking in the 60–69 years age interval (reaching approximately 400 per 100000 population in 1990 and dropping to around 300 per 100000 in 2021), before gradually tapering off among the oldest (aged ≥90 years).

When stratified by sex, the age-specific patterns remained largely similar, although the magnitude of the burden differed considerably. Across the entire age spectrum, males exhibited markedly higher age-specific DALY rates than females in both the baseline and recent years (Supplementary file Figures 1B and 1D). For instance, the peak DALYs rate for males in 1990 exceeded 500 per 100000 population (within the age group of 65–69 years), whereas the corresponding peak for females remained below 300 per 100000. Nevertheless, the dumbbell plots confirmed that both sexes successfully achieved reductions in age-specific rates across every age group over the 32-year observation period.

### Association between the burden trends and sociodemographic index

**Figure 2:**
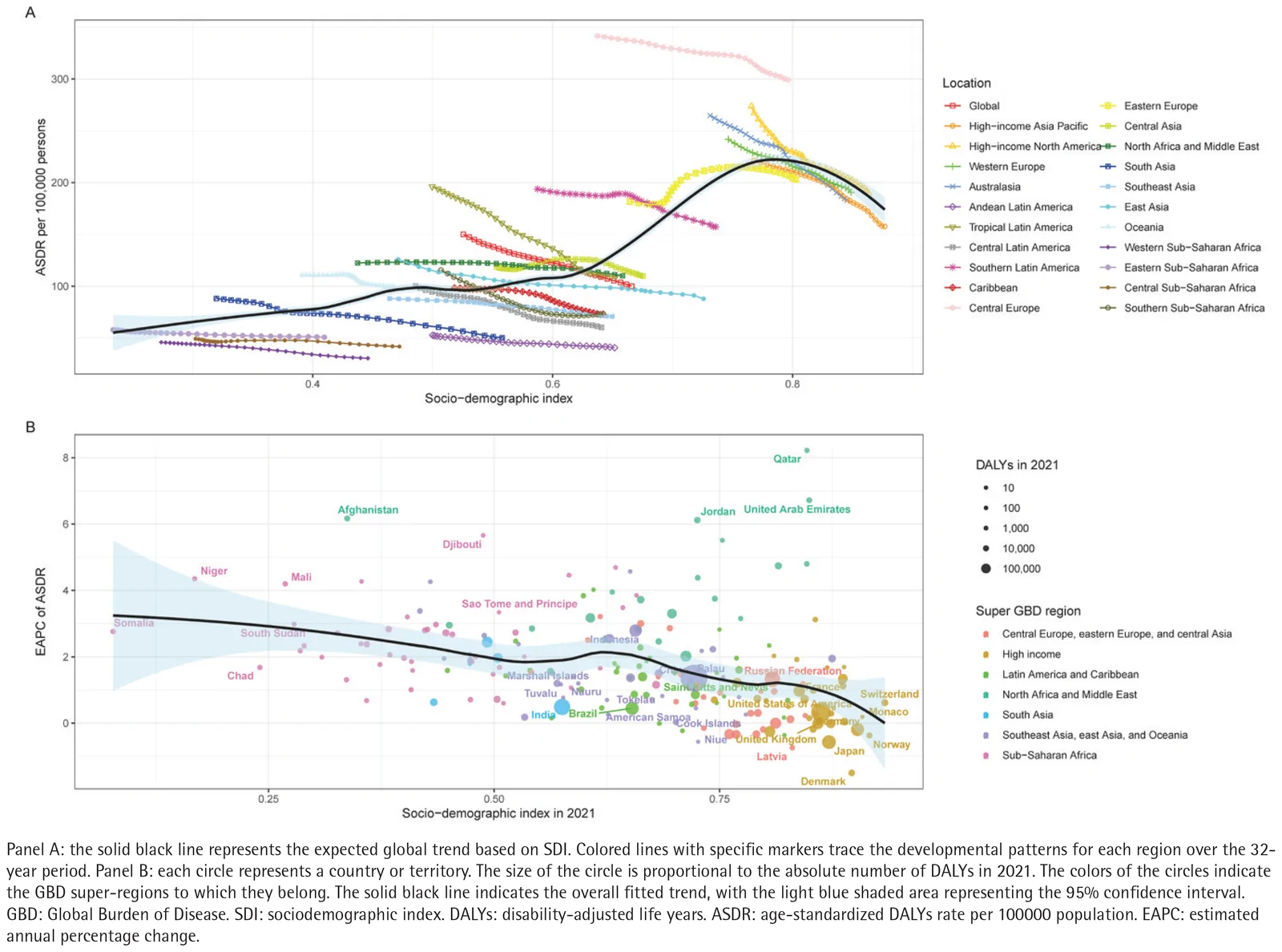
The association between SDI and ASDR and its EAPC, for low-back pain caused by smoking: A) The temporal trajectories of ASDR (age-period-cohort population) across 21 GBD regions plotted against their corresponding SDI; B) The correlation between SDI in 2021 and the EAPC of ASDR at the national level (204 countries and territories)

The spatial-temporal relationship between the burden of LBP attributable to smoking and sociodemographic development from 1990 to 2021 is illustrated in Figure 2A. At the regional level, the trajectory of the ASDR exhibited a distinct non-linear, inverted U-shaped pattern in relation to the SDI. In regions with lower sociodemographic development (SDI <0.6), the ASDR generally increased in tandem with rising SDI over the three decades. The disease burden plateaued and reached its peak at an SDI of approximately 0.75 to 0.80. Beyond this threshold, further advancements in SDI – predominantly observed in regions such as High-income North America, Australasia, and Western Europe – were accompanied by a continuous decline in ASDR, indicating that higher levels of socioeconomic development eventually mitigate the disease burden.

To further explore this developmental disparity, we assessed the correlation between the SDI in 2021 and the EAPC of ASDR across 204 countries and territories (Figure 2B). A clear negative correlation was identified (depicted by the downward-sloping trend line), demonstrating that nations with a higher SDI in 2021 tended to experience more rapid and pronounced reductions (more negative EAPCs) in their age-standardized burden over the 32-year period.

### National-level heterogeneity in disease burden trends

Despite the overall global reduction in the age-standardized burden, remarkable heterogeneity was observed in the temporal trends across the 204 countries and territories from 1990 to 2021. According to our analysis of the EAPC, while the majority of nations successfully achieved a decrease in their ASDR, a notable subset of countries experienced alarming upward trajectories (Supplementary file Table 1).

The most substantial and rapid improvements were predominantly clustered in high-SDI European countries, as well as certain nations in Latin America and the Caribbean. Specifically, Denmark achieved the most prominent global decline in ASDR, with an EAPC of -2.29 (95% CI: -2.44 – -2.14). This was closely followed by the Dominican Republic (EAPC= -2.21; 95% CI: -2.39 – -2.02), Brazil (EAPC= -2.13; 95% CI: -2.24 – -2.01), and Norway (EAPC= -1.57; 95% CI: -1.67 – -1.47). These robust downward trends indicate highly effective mitigation of the smoking-attributable LBP burden at the population level over the past three decades.

In stark contrast, several countries – primarily located in regions with lower sociodemographic development, such as Sub-Saharan Africa, North Africa, and the Middle East – exhibited concerning increases in their age-standardized burden. Afghanistan recorded the most drastic surge globally, with the ASDR increasing at an EAPC of 2.37 (95% CI: 2.19–2.56) from 1990 to 2021. Other nations demonstrating significant positive EAPC included Mali (EAPC=1.20; 95% CI: 1.04–1.35), Niger (EAPC=1.05; 95% CI: 1.00–1.09), and Djibouti (EAPC=0.67; 95% CI: 0.57–0.78).

### Age-period-cohort effects on the burden

An age-period-cohort model was utilized to evaluate the underlying temporal dynamics of the smoking-attributable LBP burden (Supplementary file Figure 2).

The local drift values were predominantly below zero across all age groups, indicating a pervasive reduction in age-specific DALYs rate for both sexes over the past 32 years (Supplementary file Figure 2A). The longitudinal age curve, reflecting the expected biological risk, displayed an inverted U-shape. The expected DALYs rate increased from the age of 30 years, peaked within the age group of 60–69 years, and subsequently declined among those aged ≥80 years. Across the entire lifespan, males consistently exhibited substantially higher expected rates than females (Supplementary file Figure 2B).

Adjusted for age and cohort biases, the period relative risks (RRs) exhibited a monotonic downward trend from 1990 to 2021 for both sexes (reference period: 2007.5) (Supplementary file Figure 2C). Similarly, the cohort RRs demonstrated a continuous and steep reduction for successively younger generations. Compared to the 1930 reference birth cohort, more recent birth cohorts faced a progressively lower risk of smoking-attributable LBP (Supplementary file Figure 2D).

### Joinpoint regression analysis of global trends

To identify specific structural shifts in the global burden, a joinpoint regression analysis was performed on the ASDR from 1990 to 2021.

Globally, the ASDR for both sexes combined decreased significantly over the 32-year period, with an AAPC of -1.29 (Supplementary file Figure 3). The model identified five distinct joinpoints (1993, 1999, 2006, 2011, and 2016), dividing the overall trend into six continuous, significantly declining segments. Notably, the most rapid reduction occurred during the most recent period, from 2016 to 2021 (APC= -1.70).

When stratified by sex, females exhibited a faster overall decline (AAPC= -1.53) compared to males (AAPC= -1.18) throughout the study period. For males, five inflection points were identified (1993, 1996, 1999, 2011, and 2016), with the steepest decrease also observed in the final segment from 2016 to 2021 (APC= -1.60) (Supplementary file Figure 4). For females, the five identified joinpoints occurred in 1993, 1996, 2006, 2011, and 2014. All trend segments for females showed significant downward slopes, with the sharpest decline occurring between 2011 and 2014 (APC= -2.15) (Supplementary file Figure 5).

## DISCUSSION

This study provides a comprehensive and up-to-date spatiotemporal analysis of the global burden of LBP attributable to smoking from 1990 to 2021. Our findings underscore a complex dual reality: while the absolute number of DALYs surged by nearly 30% primarily driven by global population growth and accelerated demographic aging, the global ASDR achieved a remarkable reduction of over 35%. Notably, advanced age-period-cohort and joinpoint regression modeling revealed continuous improvements in both historical period and generational cohort effects, accompanied by an accelerated decline in the overall burden during the most recent period (2016–2021). However, this progress remains highly unequal. The burden trajectory demonstrated a distinct inverted U-shaped relationship with sociodemographic development, revealing that while high-income nations have successfully mitigated this burden, many developing countries are facing an alarming upward trend. Collectively, these findings provide compelling real-world evidence that global tobacco control initiatives are highly effective, albeit unevenly distributed, strategies for alleviating the monumental burden of musculoskeletal diseases.

The pronounced sex and age disparities observed in our study warrant careful biological and sociological interpretations. Consistent with the global epidemiology of tobacco use, males bore more than twice the age-standardized burden compared to females. This disparity is primarily driven by the persistently higher prevalence of daily smoking among men globally, particularly in developing nations^18^. Furthermore, men are historically more likely to engage in occupationally demanding physical labor, such as heavy lifting and whole-body vibration tasks. The combination of nicotine-induced microvascular damage and heavy mechanical stress exerts a synergistic deleterious effect on the lumbar spine, accelerating intervertebral disc degeneration^19^. Regarding age dynamics, the longitudinal age curve displayed a characteristic inverted U-shape, with the biological risk peaking within the 60–69 years age bracket. This peak reflects the prolonged latency and cumulative structural damage caused by chronic smoking. Because adult intervertebral discs are highly avascular and rely heavily on diffusion for nourishment, decades of smoking-induced vasoconstriction, endothelial dysfunction, and carbon monoxide exposure lead to severe cellular malnutrition and matrix degradation, culminating in chronic disabling pain during early old age^20^. Interestingly, the expected DALYs rates subsequently declined among the oldest population (≥80 years). Rather than implying a protective effect in extreme old age, this phenomenon is largely attributable to the ‘competing risk’ of mortality, or survivorship bias^21^. Heavy smokers are significantly more likely to die prematurely from fatal cardiovascular diseases, respiratory disorders, or smoking-related cancers before reaching their eighties^22^, thereby selectively leaving a disproportionate number of non-smokers or healthier individuals in the surviving oldest demographic, which artificially lowers the smoking-attributable LBP burden in this age group.

The profound regional heterogeneity and the distinctive inverted U-shaped relationship between the SDI and the disease burden provide crucial insights into the evolving landscape of global public health. Consistent with the theory of the epidemiological transition^23^, we observed that as nations progressed from low to middle SDI levels, their age-standardized burden of smoking-attributable LBP paradoxically increased, peaking at an SDI of approximately 0.75–0.80. This escalation is largely driven by the aggressive market expansion of transnational tobacco companies into developing regions – particularly across Sub-Saharan Africa, North Africa, and the Middle East – where tobacco control legislation remains weak or poorly enforced^24^. Concurrently, rapid industrialization in these transitional economies frequently outpaces the implementation of occupational health and safety standards. Consequently, workers in these regions are increasingly exposed to the synergistic hazards of rising smoking prevalence and ergonomically poor, physically demanding labor, leading to severe musculoskeletal consequences^25^. Conversely, nations that have crossed the high-SDI threshold – such as Denmark, Norway, and High-income North America – demonstrated the most rapid and pronounced declines in their age-standardized burden. This substantial reduction (manifested as highly negative EAPCs) reflects the dividends of decades of comprehensive, multi-sectoral public health interventions. High-income countries have not only implemented stringent tobacco control measures, including exorbitant taxation, comprehensive smoke-free laws, and plain packaging^26^, but have also drastically transformed their labor markets. The transition toward service- and knowledge-based economies, coupled with widespread ergonomic improvements and advanced automation in the workplace, has substantially minimized occupational mechanical stress on the lumbar spine^27^. These divergent trajectories underscore an urgent global health inequity: the avertable burden of smoking-attributable musculoskeletal disease is being disproportionately shifted to the most vulnerable populations in developing nations.

The underlying temporal dynamics of the global burden, delineated by our joinpoint regression and age-period-cohort analyses, offer highly encouraging evidence regarding the real-world effectiveness of international tobacco control policies. Our joinpoint model revealed a continuous, global decline in the age-standardized burden over the past three decades, punctuated by a significant acceleration in the reduction rate during the most recent period from 2016 to 2021 (APC= -1.70). This accelerated decline is strongly corroborated by the monotonic downward trend observed in the period effects of our age-period-cohort model. This synchronous, cross-sectional improvement across all age groups over recent years aligns remarkably well with the intensified global implementation and maturation of the World Health Organization Framework Convention on Tobacco Control (WHO FCTC)^24^. As the most widely embraced international public health treaty, the FCTC’s MPOWER measures, particularly the widespread adoption of mass media campaigns, graphic health warnings, comprehensive bans on tobacco advertising, and the expansion of smoke-free public environments, have catalyzed a profound cultural shift, rapidly denormalizing smoking behaviors and accelerating smoking cessation rates on a global scale^26^. Equally promising is the pronounced and continuous decline observed in the cohort relative risks. We found that successively younger birth cohorts (from 1900 to the 1990s) face a progressively lower intrinsic risk of developing smoking-attributable LBP. This robust cohort-driven improvement reflects a substantial ‘generational dividend’^28^. Decades of relentless global health education and school-based prevention programs have successfully delayed the age of smoking initiation and significantly suppressed peak smoking prevalence among younger demographics^18^. Consequently, this emerging generation is accruing far less cumulative microvascular and structural damage to their intervertebral discs compared to their predecessors, foreshadowing a sustained, long-term alleviation of the LBP burden as these younger cohorts eventually age into high-risk demographic brackets.

### Limitations

Several limitations should be considered when interpreting the findings of this study. First, the inherent nature of the Global Burden of Disease (GBD) methodology means our estimates are fundamentally constrained by the availability and quality of raw empirical data^16^. In regions with underdeveloped health informatics, particularly across Sub-Saharan Africa and parts of Oceania, the reliance on complex statistical extrapolations (e.g. DisMod-MR) rather than direct primary surveillance may obscure true localized variations in the LBP burden. Second, characterizing tobacco exposure at the population level relies predominantly on self-reported survey data, which is intrinsically vulnerable to social desirability and recall biases, potentially leading to an underestimation of true smoking prevalence^29^. More importantly, the current GBD framework captures combustible tobacco use but lacks sufficient granular data to isolate the musculoskeletal impact of passive (secondhand) smoke exposure. Furthermore, the rapid global proliferation of novel nicotine delivery systems, such as e-cigarettes and heated tobacco products, poses a new challenge. Their long-term microvascular effects on spinal health remain largely unquantified in historical datasets and represent a critical gap for future epidemiological research^30^. Third, LBP is a profoundly subjective and multifactorial symptom complex rather than a uniform pathological entity^31^. Despite the GBD’s rigorous use of the MR-BRT tool to standardize conflicting data sources, significant residual confounding likely persists. Cross-national disparities in pain tolerance thresholds, cultural illness behaviors, and unequal access to diagnostic imaging mean that administrative healthcare records (e.g. ICD coding) may not be perfectly comparable across different socioeconomic settings^32^. Finally, as this is an aggregate-level ecological analysis, the observed spatiotemporal trends establish robust macro-level associations but cannot be used to infer direct causality at the individual level^33^.

### Future research

Building upon these findings and acknowledging current limitations, several critical avenues for future research and public health action emerge. First, as the global tobacco landscape rapidly evolves, future epidemiological studies must establish longitudinal cohorts to rigorously track the musculoskeletal impacts of novel nicotine delivery systems – such as e-cigarettes and heated tobacco products. Understanding whether these alternatives exert similar microvascular vasoconstrictive effects on the intervertebral discs is paramount for updating clinical guidelines. Second, to improve the fidelity of future global estimates, international consensus must be reached on standardized, culturally adapted diagnostic criteria for low-back pain, ideally complemented by objective anatomical biomarkers (e.g. standardized MRI grading for disc degeneration) incorporated into national health registries^34^. Third, from a public health perspective, the alarming upward trajectory of the LBP burden in low- and middle-income countries (LMICs) necessitates a strategic paradigm shift. International health organizations must prioritize resource allocation and legislative support to help developing nations – particularly in Sub-Saharan Africa and the Middle East – resist tobacco industry interference and fully implement the WHO FCTC MPOWER measures^35^. Finally, given the profound sex disparities and the synergistic damage caused by smoking and physical labor, future workplace wellness programs should adopt a multi-sectoral approach. Integrating targeted smoking cessation initiatives directly with occupational ergonomic interventions for high-risk populations (e.g. male manual laborers) could yield compounding benefits, significantly alleviating the global socioeconomic toll of musculoskeletal disorders^36^.

## CONCLUSIONS

Over the past three decades, the global age-standardized burden of low-backpain attributable to smoking has steadily declined, driven by robust improvements across successive periods and younger birth cohorts. However, the absolute number of affected individuals continues to surge globally due to population growth and aging. More critically, this progress is alarmingly uneven, heavily favoring high-income nations while the burden paradoxically intensifies in developing regions. Our findings underscore that smoking is not merely a cardiopulmonary hazard, but a profound, modifiable driver of global musculoskeletal disability. To mitigate the compounding socioeconomic toll of low-back pain, international stakeholders must urgently prioritize equitable, global implementation of comprehensive tobacco control policies – particularly in vulnerable, lower income nations – while integrating targeted smoking cessation programs directly into occupational health initiatives for high-risk populations.

## Supplementary Material



## Data Availability

The datasets analyzed in the current study are publicly available from the Global Health Data Exchange (GHDx) query tool (http://ghdx.healthdata.org/gbd-results-tool).
