# Supplementary material for: Global, regional, and national burden of low-back pain attributable to smoking from 1990 to 2021: An age-period-cohort and joinpoint analysis of the Global Burden of Disease Study 2021
Source: Tob Induc Dis. 2026 Sep 3;24:10.18332/tid/226563. doi: 10.18332/tid/226563 (PMC13537002; doi:10.18332/tid/226563)
Supplement: Supplementary file 1 [file TID-24-153-s1.pdf]

Tobacco Induced Diseases

Supplementary file

© 2026 Kou J. et al.

DOI:
10.18332/tid/226563

The content has been provided by the author(s) and has not been reviewed, verified, or endorsed by European Publishing. It may not have undergone peer review. The views, opinions, and recommendations expressed are solely those of the author(s) and do not necessarily reflect the position of European Publishing. European Publishing accepts no responsibility or liability for any consequences arising from the use of, or reliance on, this content.

Global, regional, and national burden of low back pain attributable to smoking from 1990 to 2021: An age-period-cohort and Joinpoint analysis of the Global Burden of Disease Study 2021

Table of Contents

1. Supplementary Table 1. The absolute DALYs, age-standardized DALY rates (ASDR), and estimated annual percentage changes (EAPC) of low back pain caused by smoking across 204 countries and territories, 1990–2021. 2
2. Supplementary Figure 1. Sex-specific trends in the proportion and age-specific DALY rates of low back pain caused by smoking, 1990–2021. 15
3. Supplementary Figure 2. Age-period-cohort effects on the DALY rates of low back pain caused by smoking by sex. 16
4. Supplementary Figure 3. Joinpoint regression analysis of the age-standardized DALY rates for low back pain caused by smoking globally (Both sexes), 1990–2021. 17
5. Supplementary Figure 4. Joinpoint regression analysis of the age-standardized DALY rates for low back pain caused by smoking globally (Male), 1990–2021. 18
6. Supplementary Figure 5. Joinpoint regression analysis of the age-standardized DALY rates for low back pain caused by smoking globally (Female), 1990–2021....19

1. ***Supplementary Table 1. The absolute DALYs, age-standardized DALY rates (ASDR), and estimated annual percentage changes (EAPC) of low back pain caused by smoking across 204 countries and territories, 1990 －2021***

Location	1990 DALYs (95% UI)	1990 ASDR (95% UI)	2021 DALYs (95% UI)	2021 ASDR (95% UI)	1990 – 2021 Trend EAPC (95% CI)
Afghanistan	2105.6 (981.7-3726.9)	29.3 (13.7-52.1)	10587 (5235.1-17980.8)	54.9 (27.1-94)	2.37 (2.19-2.56)
Albania	6188.3 (3348.2-9390.4)	240.5 (126.7-360.5)	9707.9 (5302.3-15345.3)	263.1 (148-419.4)	0.43 (0.34-0.51)
Algeria	16274.4 (8624.7-25063.3)	109.8 (57.8-166.2)	39053.1 (21166.8-61190.8)	89.1 (48-140.1)	-0.73 (-0.78--0.69)
American Samoa	36.8 (19.3-58.8)	112.5 (58.7-179.2)	48.4 (24.3-78.6)	91.2 (45.6-148.3)	-0.84 (-0.96--0.72)
Andorra	149.4 (80.4-223.9)	235.5 (127.8-354.9)	273.9 (150.6-434.5)	212.3 (117-335.1)	-0.33 (-0.35--0.32)
Angola	4546.4 (2306.8-7558.6)	81.9 (41.8-135.8)	10010.6 (5154.2-17111.6)	52.9 (27.1-91.1)	-1.41 (-1.49--1.33)
Antigua and Barbuda	30.2 (13.8-57.2)	59.9 (27.7-113.1)	73.2 (34.4-133.3)	64.6 (30.2-117.9)	0.18 (0.16-0.2)
Argentina	61348.8 (33057.2-97151.3)	190.9 (103.1-301.7)	78425.5 (40898.6-129678.8)	148.6 (77.9-245.1)	-0.79 (-0.9--0.68)
Armenia	5172.6 (2992.1-7750.9)	165.8 (95.7-247.5)	6501.9 (3600.9-9832.4)	156.8 (88.3-236.8)	-0.11 (-0.16--0.06)
Australia	49493.6 (26555-74637.8)	263 (141.6-394.6)	62892.6 (30629.5-97700.6)	185.7 (89.8-289.7)	-1.11 (-1.17--1.04)
Austria	18428.8 (9957.3-29075.9)	196.7 (107.9-302.1)	24399.5 (13304.7-37356.4)	188.4 (101.9-287.1)	-0.01 (-0.1-0.08)
Azerbaijan	6615 (3537.1-9647.7)	114.1 (61.5-166.7)	15596.9 (9049.3-24165.2)	128.6 (74.9-199.5)	0.43 (0.27-0.6)
Bahamas	84.7 (40.2-146.6)	42.7 (20.3-73.4)	180.8 (86.5-342.5)	38.3 (18.1-72.7)	-0.41 (-0.48--0.34)

Bahrain	424.1 (212.2-676.5)	115.6 (57.9-175.6)	1806 (972.8-2949.2)	103.3 (55.1-167.3)	-0.22 (-0.35--0.09)
Bangladesh	78397.6 (43740.7-118434.3)	135.5 (73.9-205.8)	155302.7 (84994.5-239847.6)	108.4 (59.7-167.6)	-0.63 (-0.71--0.55)
Barbados	129.3 (63.1-225.3)	51.4 (25.4-90.1)	170.3 (80-328.7)	41.9 (19.3-81.7)	-0.97 (-1.11--0.83)
Belarus	22677.4 (12880.2-34832)	187.4 (106-283)	25268.5 (13959.2-39813.3)	183.8 (99.9-287.9)	0 (-0.12-0.12)
Belgium	31668.2 (17543.9-48074.7)	250.9 (141.1-378)	30110.6 (14209.5-46497.3)	188.8 (88.7-291.2)	-0.93 (-0.98--0.88)
Belize	71.3 (35.7-118.9)	68.4 (34.6-113.4)	215.9 (106.2-374)	61.2 (30.2-104.8)	-0.12 (-0.22--0.02)
Benin	1151.3 (547.9-1877.3)	47.4 (23.3-77.2)	2200.1 (1061.2-3733.2)	30.8 (14.8-52.4)	-1.71 (-1.94--1.47)
Bermuda	63.8 (32.3-102.5)	92.7 (47.7-148.9)	72.7 (37.5-131.7)	72.1 (36.8-131.1)	-1.02 (-1.12--0.92)
Bhutan	246.4 (114.1-422.8)	74 (34.5-124.2)	429.6 (209.1-790.5)	59.6 (28.9-108.9)	-0.75 (-0.78--0.72)
Bolivia (Plurinational State of)	1758.4 (801.7-2909.8)	44.2 (20.8-72.4)	4746.8 (2467.9-7994.6)	43.7 (22.8-74.4)	0.46 (0.22-0.7)
Bosnia and Herzegovina	12642 (7100.6-19490.1)	266.1 (147.6-410.4)	14917.5 (7899-23038)	312.4 (165.7-468.5)	0.63 (0.42-0.84)
Botswana	632.2 (332.3-982.1)	88.3 (46.9-138.4)	1827.6 (963.6-2951.2)	92.9 (49.5-149)	0.23 (0.21-0.26)
Brazil	222128.1 (127121.9-338512.4)	197.3 (111.4-304.2)	278668.7 (139740.9-438614)	109.3 (54.8-172)	-2.13 (-2.24--2.01)
Brunei Darussalam	303.5 (162.7-483.4)	167.2 (89.5-257.1)	575.9 (292.8-961.8)	111.9 (56.9-187)	-1.25 (-1.38--1.12)
Bulgaria	37127.2 (20543.3-57305.2)	340 (189.9-516.8)	36432.9 (19591-54485)	350 (191.7-514.3)	0.04 (-0.02-0.09)
Burkina Faso	2327.9 (1159.5-3652.7)	43.8 (21.8-68.5)	4533.4 (2249.6-7674.2)	34 (17.1-56.6)	-0.91 (-0.95--0.87)
Burundi	1847.6 (901.7-3016.8)	62.3 (30.7-102.3)	3421.9 (1717.4-6148.2)	47.9 (24.4-84.8)	-0.64 (-0.78--0.5)

Cabo Verde	98.1 (48.5-165.3)	48 (23.7-80.5)	179 (88.5-316.6)	37.8 (18.7-67)	-0.77 (-0.83--0.72)
Cambodia	5764.4 (3276.7-8728.9)	104.2 (57.4-156.8)	11424.8 (5897.5-17574.4)	76 (39.7-117.2)	-1.37 (-1.53--1.22)
Cameroon	3627.4 (1871.1-5628.5)	61.4 (31.5-95.5)	6926.5 (3551-12302.9)	37.6 (19.3-66.2)	-1.7 (-1.91--1.49)
Canada	82540.4 (43643.8-124664.3)	259.3 (138-387.6)	88719.2 (44499.9-137211.6)	169.5 (84.7-259.8)	-1.53 (-1.62--1.44)
Central African Republic	822.6 (407-1356.2)	53.7 (26.9-88.1)	1495.9 (748.2-2583.3)	43.3 (21.5-75.1)	-0.73 (-0.84--0.62)
Chad	2166 (1064.5-3584.6)	65.7 (32.4-108.9)	3764.3 (1866.9-6574.9)	46.7 (22.9-81.1)	-1.28 (-1.49--1.07)
Chile	23798.2 (13352.6-36492.3)	201.8 (113.9-309.7)	39634.1 (19297.2-64906.1)	172.8 (83.7-280.4)	-0.47 (-0.53--0.41)
China	1255932.7 (742462.8-1918254)	126.4 (73.9-193.1)	1792215.6 (998595.6-2722182.7)	87.3 (48.5-131.5)	-0.98 (-1.07--0.9)
Colombia	26001.3 (13730.4-40454.2)	112.3 (58.6-175)	34852.9 (16578.7-59120.1)	62.1 (29.5-105.6)	-1.86 (-1.96--1.76)
Comoros	151.9 (76.8-236.9)	60 (30.4-94.9)	278.1 (139.6-464.8)	46.2 (23.1-76.6)	-1 (-1.09--0.9)
Congo	578.2 (291.9-969.4)	42.9 (21.4-71.4)	2046.4 (1025.3-3432.7)	53 (26.7-91.1)	0.92 (0.8-1.03)
Cook Islands	19.7 (10.6-29.8)	133.4 (71.9-204.2)	21.5 (11.1-35.8)	115.6 (60-195.2)	-0.52 (-0.61--0.44)
Costa Rica	2320.4 (1199.2-3688.7)	107.1 (56.8-171)	3766.6 (1857.2-6325.9)	64.1 (31.6-107.5)	-1.55 (-1.74--1.36)
Coted'Ivoire	3800.8 (1802-6061.3)	58.6 (28.7-95.6)	9572.6 (4890.4-16409.6)	49.4 (25.3-85.1)	-0.6 (-0.79--0.41)
Croatia	21537.9 (12087.5-32490.6)	351.7 (198.7-523.1)	18892.6 (10220.4-28687.3)	323.9 (179.7-491.2)	-0.05 (-0.12-0.03)
Cuba	13907.2 (7441.1-22015.1)	133.8 (71.8-210.7)	19266.2 (9836.3-32514.4)	115.7 (58.7-194.3)	-0.58 (-0.69--0.47)

Cyprus	1893.9 (1045.1-2926.2)	235.8 (130-362.2)	3545.7 (1942-5590.5)	193 (106.2-304.3)	-0.7 (-0.79--0.6)
Czechia	45316.2 (25536.5-69547.4)	363.2 (204.7-558.4)	46759.3 (26047.8-73129.8)	301 (168.4-467.2)	-0.68 (-0.71--0.65)
Democratic People's Republic of Korea	19061.4 (10796.5-28144.4)	96.9 (54.2-144.5)	29377.9 (16066.6-47258.1)	86.6 (47.3-139.4)	-0.51 (-0.6--0.42)
Democratic Republic of the Congo	8678.4 (4304.9-14229.2)	41.1 (20.6-67.3)	19690.8 (10135-33144.2)	36.5 (19-61.7)	-0.23 (-0.37--0.08)
Denmark	27811.8 (15377.6-40536)	420.4 (235.4-612.5)	19545.8 (10081.2-30003)	229.5 (116.3-355.3)	-2.29 (-2.44--2.14)
Djibouti	161.6 (79.9-255.6)	76.1 (38.3-120.8)	858 (443.2-1329.7)	87.7 (44.4-138.7)	0.67 (0.57-0.78)
Dominica	36 (16.9-65)	59.7 (28.5-107.4)	56.1 (27.9-99.5)	66.9 (33.4-118.5)	0.45 (0.3-0.59)
Dominican Republic	4198.5 (2199.9-6700.5)	94.1 (48.4-150)	5506.1 (2741-9543)	52.3 (26.2-91.2)	-2.21 (-2.39--2.02)
Ecuador	4450.8 (2299.2-7046.9)	69.3 (36-108.4)	7142.2 (3769.7-12062.5)	41.2 (21.7-69.7)	-1.49 (-1.6--1.37)
Egypt	38476.2 (21378.5-58600.5)	106 (58.1-160.9)	100588.5 (58629.8-152363.8)	116.7 (67.1-177.2)	0.35 (0.24-0.46)
El Salvador	2046.5 (983.2-3656.5)	59.6 (28.8-106.1)	2182.7 (1004.2-4364.2)	36.3 (16.7-72.7)	-1.63 (-1.71--1.54)
Equatorial Guinea	125.7 (65-199.5)	50.7 (25.9-81.4)	378.6 (202.3-601.7)	45.2 (24.1-73.7)	-0.3 (-0.35--0.24)
Eritrea	595.1 (302.8-934.3)	31.4 (15.9-49.9)	1411.3 (702.1-2356.6)	30.1 (14.9-50)	-0.13 (-0.19--0.08)
Estonia	4267.7 (2352-6595.5)	228.3 (124.5-348.9)	4224.9 (2059.5-6625.6)	220.9 (110.8-346.6)	0.13 (-0.05-0.31)
Eswatini	171.3 (86.1-284.7)	45.3 (22.5-77.1)	255.5 (120.5-435.2)	30.6 (14.6-53)	-1.3 (-1.51--1.09)
Ethiopia	8427.2 (4136.4-13606.6)	31.4 (15.4-50.3)	16145.8 (7917.4-29380.4)	26.4 (13.1-47.6)	-0.26 (-0.46--0.06)

Fiji	598.3 (308.7-922.3)	111.2 (57.3-170.6)	783.9 (427.6-1297.5)	84.1 (45.8-141.3)	-1.04 (-1.14--0.95)
Finland	11923.8 (6682.9-17772.6)	190.9 (106.7-282.8)	11826 (6269.3-17755.3)	153.3 (79.3-227.7)	-0.59 (-0.65--0.54)
France	142383.8 (75438.9-216028.3)	207.3 (111.6-312.4)	187736.7 (96559.3-292349.4)	204.1 (105-306.9)	0.05 (-0.01-0.1)
Gabon	206 (98.5-362.5)	32.4 (15.7-56.3)	766.9 (391.9-1310.7)	53.5 (27.2-92.6)	2 (1.8-2.2)
Gambia	394.2 (209.6-614.8)	80.2 (42.2-126)	699.3 (375.5-1133.7)	50.2 (26.6-82.4)	-1.7 (-1.77--1.63)
Georgia	7890.4 (4383.8-11853.4)	129.7 (72.8-193.5)	7334.5 (4161.7-11238.5)	151 (84.7-229.7)	0.64 (0.54-0.74)
Germany	297411.7 (164102.7-452304.1)	287.8 (161.6-433.7)	276957.8 (146662.4-426740.9)	225.7 (120.1-342.2)	-0.67 (-0.75--0.59)
Ghana	3140.1 (1505.5-5245.5)	38.6 (18.4-64.1)	5255.6 (2556.8-9128)	24.7 (12.1-42.4)	-1.56 (-1.62--1.49)
Greece	30707.1 (17110.7-46192.7)	238.4 (135.9-354.3)	38240.1 (20275.6-57358.4)	237.7 (130.3-346.5)	0.07 (-0.03-0.16)
Greenland	135 (74.4-212.3)	257.5 (144.1-394.7)	166.4 (93.3-258.3)	235 (131.2-364.4)	-0.35 (-0.47--0.23)
Grenada	40.8 (19.4-73.2)	58.7 (27.7-105.1)	71.5 (34.7-132.7)	53 (25.4-98.1)	-0.34 (-0.44--0.25)
Guam	126.8 (65.5-202.8)	112 (58.9-181.5)	209.7 (112.3-326.2)	109.7 (57.7-169.2)	-0.06 (-0.1--0.02)
Guatemala	3943.9 (1977-6631.3)	85.5 (42.9-144.8)	6167 (2849.2-11244.3)	48.5 (22.6-88.4)	-1.76 (-1.84--1.69)
Guinea	2407.7 (1190-4069.2)	63.5 (31.2-106.9)	3909.7 (1956.2-6763.6)	52.5 (26.1-91.4)	-0.72 (-0.83--0.61)
Guinea-Bissau	117.2 (55-212.1)	22 (10.4-38.8)	388.3 (194-667.2)	32.7 (16.5-57.4)	1.56 (1.44-1.69)
Guyana	307.7 (156.7-489.8)	61.3 (31.1-99.4)	471.5 (245.5-796.1)	60.1 (31.3-102.3)	-0.09 (-0.16--0.02)
Haiti	2218.9 (1122-3656.7)	52.9 (26.7-87.5)	3746.3 (1813.5-7305.2)	37.2 (18.2-73.7)	-1.25 (-1.4--1.11)

Honduras	2182.9 (1109.3-3661.7)	83.7 (42.5-138.7)	3617.4 (1763.8-6002)	45.5 (22.3-75.8)	-2.2 (-2.36--2.05)
Hungary	49006.5 (28138-74639.2)	377.2 (216.5-573.7)	42508.4 (22534.6-66287.8)	307.6 (164.5-474.9)	-0.6 (-0.7--0.5)
Iceland	623.2 (351.9-971.9)	237.9 (134.7-368.8)	751.1 (391.6-1248.4)	157.2 (82.8-261.4)	-1.44 (-1.49--1.4)
India	455603.1 (243499.2-716074.6)	78.2 (41.9-123.1)	511424.5 (251628.4-834774.1)	39.1 (19.6-63.7)	-2.14 (-2.33--1.96)
Indonesia	101390.6 (57950.4-153308.3)	78.9 (45-118.5)	233968.6 (126963.3-348474.4)	78.8 (42.4-117.8)	0.05 (-0.06-0.16)
Iran (Islamic Republic of)	38172.3 (19526.2-61217.9)	112.3 (57.2-176)	100210.8 (51138.3-165630.9)	100.3 (51.5-167.6)	-0.3 (-0.38--0.22)
Iraq	13034.8 (7029.9-20042)	131 (69-200.9)	38553 (21810.7-59969.2)	119.9 (68.1-187)	-0.22 (-0.3--0.14)
Ireland	9625.1 (5039.2-14482.8)	257 (135.5-387.3)	10678.6 (5623-16791.7)	156.6 (81.7-245.5)	-1.75 (-1.88--1.63)
Israel	10224.3 (5718.9-16059.3)	220.7 (124.8-346.6)	16220.4 (8285.8-26065.7)	160.2 (82.8-257.1)	-1.04 (-1.09--0.99)
Italy	168545.6 (90394.4-254594.3)	233.4 (124.2-348.6)	154083.8 (82665.5-244295.2)	169.4 (90.4-266.8)	-1.04 (-1.11--0.97)
Jamaica	1525.2 (800.3-2394.2)	89.2 (46.8-138.4)	1706.6 (831-2983.5)	55.8 (27.1-97.6)	-1.81 (-1.99--1.63)
Japan	376126.7 (208961.4-560471.3)	237 (132.3-350.3)	316564.5 (168560.6-516220.8)	170.7 (91.3-276.1)	-1.06 (-1.15--0.96)
Jordan	3086 (1741.4-4650.1)	160.9 (88.6-245.9)	18046.5 (10317.7-27293.7)	167.8 (97.2-256.8)	0.18 (0.12-0.23)
Kazakhstan	19138.8 (10831.7-29515.9)	127.5 (71.4-195.5)	26334.5 (13147.9-40669.6)	123.1 (61.4-190.1)	-0.08 (-0.27-0.11)
Kenya	6701.1 (3395.8-10615.4)	64.1 (32-101.8)	16069.4 (8589.3-25893.1)	50.7 (26.4-82.7)	-0.9 (-0.98--0.83)

Kiribati	73 (40.6-111.6)	152.8 (85.9-233.1)	157.6 (87.8-242.9)	161.7 (90.4-246.3)	-0.01 (-0.23-0.21)
Kuwait	1682.9 (930.1-2626.4)	131 (71-197.4)	6080.7 (3290.2-9501.2)	111 (59.6-176.1)	-0.6 (-0.64--0.56)
Kyrgyzstan	4059.8 (2312.2-6176.3)	123.9 (69.7-188.9)	8107.9 (4368.1-12164.1)	126.9 (67.7-190.4)	0.33 (0.16-0.51)
Lao People's Democratic Republic	2439.5 (1309.2-3745.9)	96.7 (51.7-147.5)	5211.6 (2957.9-8161.4)	85.1 (48.6-132.6)	-0.3 (-0.37--0.23)
Latvia	6982.1 (3891.1-10605.1)	212.1 (117.5-319.5)	5347.9 (3085.2-8190.7)	192.3 (107.5-292)	-0.13 (-0.26-0)
Lebanon	3536.7 (1850.7-5440.9)	140.1 (72.7-215.9)	11047 (6095.4-17236.8)	186.5 (103-291.9)	0.94 (0.65-1.23)
Lesotho	551.4 (278-882.8)	58.6 (29.5-95.5)	700.7 (382.6-1085.5)	49 (26.6-76.4)	-0.61 (-0.73--0.49)
Liberia	700.9 (354.8-1129.4)	50.1 (25.3-79.1)	1348.2 (661.3-2324.1)	38.3 (18.9-66.7)	-0.99 (-1.13--0.86)
Libya	2597.7 (1417.2-4004.2)	105.1 (56.2-162.3)	8371.7 (4717.4-12725.1)	111 (62.3-169.1)	0.42 (0.3-0.55)
Lithuania	8007.6 (4513.1-12400.4)	192.5 (108.9-296.5)	8448 (4414.1-12973.2)	210.2 (112-315.6)	0.54 (0.46-0.62)
Luxembourg	1128.6 (618.4-1758.1)	232.1 (128.2-360.2)	1652.6 (893.3-2648.5)	187.9 (102.2-296.7)	-0.75 (-0.81--0.69)
Madagascar	5045.9 (2707.7-7721.1)	77.3 (41.1-119.3)	8786.8 (4891-13915.7)	49.9 (27-78.9)	-1.34 (-1.59--1.09)
Malawi	3183.6 (1636.7-5038.2)	67.9 (34.8-109.6)	5876 (3025.9-9570.3)	59.6 (30.6-97.9)	-0.42 (-0.46--0.38)
Malaysia	9172.9 (5013-13667.8)	74.4 (40.4-109.5)	20000.1 (10900.3-31150)	57.3 (31.4-90.5)	-1.04 (-1.15--0.92)
Maldives	109.8 (62-163.6)	97.5 (54.5-144.9)	421.3 (229.5-681.7)	81.3 (44.4-131)	-0.65 (-0.79--0.5)
Mali	1615.7 (796.2-2621.3)	31.9 (15.7-52)	5288.7 (2603.6-8994.1)	44.1 (21.8-74.4)	1.2 (1.04-1.35)

Malta	1097.2 (608.1-1663.2)	254 (141.5-383)	1374.7 (731.1-2176.2)	183.7 (99.3-290.4)	-1 (-1.1--0.9)
Marshall Islands	19.9 (10-32.4)	83.1 (41.7-131.7)	29.2 (15.7-47.7)	78.7 (41.5-128.8)	-0.19 (-0.21--0.18)
Mauritania	648.4 (330.3-1036)	51.9 (26.7-83)	1088.2 (546.9-1823.8)	38.9 (19.7-65.4)	-1.12 (-1.21--1.02)
Mauritius	742.4 (397.3-1130.2)	81.3 (43.3-121.9)	1076.8 (591.6-1733.8)	62.9 (34.5-102.2)	-0.7 (-0.81--0.6)
Mexico	54047.7 (29063.3-85217.2)	98.9 (53.7-155.9)	87793.1 (43402.9-136794.9)	63.3 (31.3-98.5)	-1.56 (-1.7--1.42)
Micronesia (Federated States of)	86.8 (46.7-132)	142.4 (78.6-220.3)	133 (74.8-212.6)	140.2 (78.6-222)	-0.04 (-0.12-0.04)
Monaco	106.4 (53.6-169.4)	231.4 (117.2-371.4)	114.5 (60.1-191.1)	190.8 (103.7-321.7)	-0.67 (-0.7--0.64)
Mongolia	1798.1 (984.8-2769.9)	142.2 (75.9-220.6)	4566.8 (2438.5-6965.8)	143.4 (76.4-219.4)	0.08 (0.04-0.12)
Montenegro	2340.9 (1315.4-3645.6)	359.8 (200.3-559.9)	3206.1 (1770.2-4905.3)	363.7 (202-542.5)	0.07 (-0.03-0.18)
Morocco	18133.1 (9775.6-27363.5)	103 (54.6-157.6)	30255.6 (16761.5-49081.6)	76.8 (42.5-124.7)	-1.03 (-1.09--0.97)
Mozambique	3798.9 (1890.9-6242.4)	48.5 (24-79.6)	8216 (4201.9-13717.7)	52.4 (27-85.1)	0.32 (-0.04-0.69)
Myanmar	27078.6 (14864.7-40671.8)	99.6 (54.2-149.5)	30338.4 (15784.4-47573.9)	55 (28.6-85.8)	-2.09 (-2.22--1.95)
Namibia	699.9 (342.1-1122.7)	89.3 (43.4-145.4)	1263.4 (638.2-2128.1)	65.5 (33.4-110.4)	-1.14 (-1.23--1.05)
Nauru	9.6 (5.2-14.8)	153.1 (84.3-238)	12.7 (7-19.2)	146.6 (79-222.9)	-0.15 (-0.22--0.08)
Nepal	30590.2 (17239.2-46381.6)	263.2 (146.9-400.3)	39978.8 (22624.4-63534.6)	149.2 (84.6-237.5)	-2.1 (-2.32--1.88)
Netherlands	43754 (24303.5-65123.9)	245.1 (137-362.5)	43949.1 (22257.7-67673.1)	172.5 (86.7-261.1)	-1.04 (-1.12--0.96)
New Zealand	10119.5 (5283.5-15565.6)	273.1 (143.1-417.1)	11019 (5442.7-17037.2)	168.6 (83.3-257.9)	-1.58 (-1.64--1.53)

Nicaragua	1628 (792.6-2903.2)	77.6 (38-137.1)	3273.3 (1581.8-5718.7)	54.5 (26.6-95.4)	-0.9 (-1--0.79)
Niger	1084.6 (524.5-1830.6)	26 (12.5-44.3)	4077.2 (1827.6-7360.7)	35.5 (16.4-63.4)	1.05 (1-1.09)
Nigeria	21418.1 (10096.1-38247.9)	40.4 (18.8-71.9)	27622 (12571.5-50724.9)	21.1 (9.7-38.5)	-2.18 (-2.32--2.04)
Niue	1.9 (1-3)	93.3 (48-148.8)	1.7 (0.9-2.9)	85.9 (44-145.4)	-0.36 (-0.44--0.29)
North Macedonia	6330.7 (3511.6-9857.6)	305.4 (169.7-473.2)	8346.4 (4507.9-12781.7)	319.1 (177.8-482.1)	0.18 (0.13-0.23)
Northern Mariana Islands	43.9 (21.8-72.4)	116.7 (60-193)	61.1 (32.5-101.2)	100.7 (53.7-168.2)	-0.53 (-0.58--0.49)
Norway	11679.5 (6696.6-18403.9)	229.9 (133.1-363)	11131.2 (5480.1-18164.2)	153.1 (74.7-248.9)	-1.57 (-1.67--1.47)
Oman	1347.7 (708.2-2252.4)	102.2 (52.9-163.9)	3278.6 (1689.3-5431.2)	66.9 (34.5-111.1)	-1.15 (-1.4--0.9)
Pakistan	62362.7 (33698-98454)	99.1 (53.4-156.5)	112746.6 (60376.7-195996.5)	74.4 (39.9-125.4)	-0.95 (-1.04--0.87)
Palau	11.6 (5.8-18.6)	93.3 (46.9-149.2)	21.1 (10.9-35.2)	86.8 (44.8-145.3)	-0.33 (-0.4--0.27)
Palestine	1279.7 (682.3-1958)	123.2 (65.3-192.5)	4427.9 (2430.8-6789.1)	122.2 (67-187.8)	-0.15 (-0.2--0.1)
Panama	1324.3 (627.3-2251.8)	75.6 (35.8-127.9)	1400.5 (626-2404.4)	32.8 (14.7-56.2)	-3.03 (-3.2--2.86)
Papua New Guinea	2742.8 (1449.3-4228.8)	106.1 (54-165.3)	7592.1 (3981-11828.8)	96.6 (50.5-154.1)	-0.39 (-0.46--0.33)
Paraguay	3774.3 (1951.2-5810.6)	144 (74.2-219.4)	6522.1 (3451.2-11041.7)	108.9 (57.7-184.7)	-1.04 (-1.12--0.95)
Peru	6858.7 (3480.6-11604.9)	47.4 (24.1-79.3)	14573.3 (7411-24354.8)	39.3 (20-66)	-0.83 (-0.92--0.73)
Philippines	47714.1 (26453.3-72262.2)	124.9 (70.2-189.2)	75470.6 (38768.4-123695.3)	77.8 (39.7-126.3)	-1.64 (-1.79--1.48)

Poland	168461.5 (93468.3-249268.7)	379.7 (212.5-558.5)	166293.6 (85033.4-249691.2)	294.7 (150-441.6)	-0.81 (-0.86--0.76)
Portugal	23818.4 (12518.6-36561.9)	200.6 (105.4-311.4)	28337.4 (14902.4-43083.1)	189.8 (100-286.1)	-0.07 (-0.13--0.01)
Puerto Rico	2869.4 (1408.6-4752.2)	79.4 (38.9-132.4)	3703.1 (1730.7-6063.4)	71 (34.2-114.6)	-0.4 (-0.43--0.38)
Qatar	416.5 (226.6-657.5)	102.4 (53.1-163.8)	3558.5 (1844.8-5777.8)	96.6 (50.8-157.5)	-0.08 (-0.18-0.03)
Republic of Korea	70919.8 (41138.5-107986.6)	171.7 (97.6-262.8)	108465.4 (56854.8-176365.2)	139.7 (73.1-224.3)	-0.68 (-0.73--0.62)
Republic of Moldova	6837 (3798.8-10777.8)	145.7 (81.4-228.6)	8801.8 (4839.8-13648.2)	166.8 (91.1-255.8)	0.47 (0.42-0.52)
Romania	80367.2 (44228.5-125024.4)	297.8 (163.7-460.6)	75399.6 (40953.9-115280.6)	273.3 (152-414.8)	-0.43 (-0.51--0.36)
Russian Federation	305213.3 (164620-457203.3)	172.6 (92.6-255.8)	447638.2 (234013.9-676362)	208.5 (111.1-313.9)	0.79 (0.63-0.95)
Rwanda	3333.4 (1627.6-5407.7)	95.5 (47.3-153.8)	7412.2 (3641-12477.6)	93.5 (45.7-161.1)	-0.09 (-0.27-0.09)
Saint Kitts and Nevis	15.4 (7.3-28.6)	48 (22.7-87.8)	21.2 (9.8-40.7)	35.4 (16.4-68.7)	-1.21 (-1.33--1.09)
Saint Lucia	69.8 (34.5-119.4)	75 (37-128.9)	142.1 (73.4-265.5)	62.5 (32.3-117)	-0.93 (-1.1--0.75)
Saint Vincent and the Grenadines	42.5 (21.6-71)	58.2 (29.6-96.9)	70.9 (36-129.4)	51 (25.5-93)	-0.44 (-0.47--0.42)
Samoa	141.3 (72.7-218.3)	139.4 (71.8-215.5)	222.6 (122.1-349.5)	128.3 (70.3-202.4)	-0.51 (-0.62--0.4)
San Marino	58.4 (32.3-93.1)	225.8 (125-361.3)	93.3 (49.7-159.8)	171.6 (93.2-293.1)	-1 (-1.06--0.93)
Sao Tome and Principe	16.9 (8.3-29.7)	24.6 (12-42.9)	44.4 (21.5-79)	28.5 (13.7-51.7)	0.41 (0.15-0.67)
Saudi Arabia	6275.3 (3160.6-10189.7)	61.1 (30.8-101.4)	28374.6 (14580.8-44546.4)	83.7 (43.7-132.6)	0.77 (0.62-0.92)

Senegal	2453.2 (1328.4-3831.9)	59.9 (32.3-93)	3662.8 (1795-5884.3)	34.1 (16.8-54.9)	-2.04 (-2.19--1.88)
Serbia	38466.5 (21970.8-57297.8)	347.7 (198.3-519)	44145.3 (23086.8-67007.1)	345.9 (181.2-522.3)	-0.05 (-0.14-0.05)
Seychelles	44.3 (23.1-70.6)	77.5 (40.4-122.7)	88.8 (47.4-146.8)	69.4 (36.5-114.2)	-0.48 (-0.56--0.41)
Sierra Leone	2182 (1158-3354.4)	83.6 (43.6-127.4)	2733.2 (1512.2-4408.9)	55.4 (30.5-90.3)	-1.35 (-1.4--1.29)
Singapore	2689.2 (1364.8-4338.7)	86.4 (43.5-139.1)	6227 (2941.4-10435.3)	72.7 (34.3-122.8)	-0.22 (-0.33--0.12)
Slovakia	15832.2 (8675.5-24361.1)	275.6 (152.1-420.2)	20337.7 (11001.2-31694.3)	262.2 (143.7-409.9)	-0.03 (-0.08-0.02)
Slovenia	6104.6 (3246.2-9625.6)	260.3 (138.6-407)	8231.2 (4289-13021.1)	268.2 (141.9-412.8)	0.3 (0.18-0.42)
Solomon Islands	231.4 (126.9-362.3)	125 (68.6-191)	787.7 (429.3-1254.3)	134.5 (72.4-215.8)	0.44 (0.34-0.53)
Somalia	2159.1 (1063.5-3684.5)	52.2 (26.1-91)	4856.1 (2413.1-8338.1)	46 (23.1-77.4)	-0.41 (-0.42--0.4)
South Africa	30750.2 (16926.5-47369.9)	123.9 (66.8-192.6)	44803.9 (23218.5-69889.3)	75.1 (38.8-117.5)	-1.52 (-1.77--1.27)
South Sudan	915.6 (427.4-1561.6)	31.6 (14.9-54.1)	2057 (1007-3531.6)	36.1 (17.8-62.5)	0.63 (0.55-0.72)
Spain	86458.5 (48098.8-126491.2)	195.5 (109-283.6)	108551.4 (55642-166624.9)	156.6 (80.6-236.3)	-0.35 (-0.47--0.22)
Sri Lanka	9403.5 (5241.2-14595.4)	70.8 (38.5-108.2)	9553.2 (4688.5-15046.7)	36.7 (18-57.2)	-2.12 (-2.16--2.07)
Sudan	6205.4 (3070.9-10201)	52.4 (26.1-84.4)	15537.5 (7851.5-26817.9)	54.8 (27.8-94.4)	0.07 (-0.08-0.21)
Suriname	306.4 (166.8-493.9)	103.4 (56.3-166.5)	539.7 (282.2-931)	81.8 (42.7-140.9)	-0.92 (-1.06--0.78)
Sweden	15083.2 (8234.7-24592)	129.1 (71.8-204.2)	21401 (10703.9-34122.6)	142.1 (70.5-228.2)	0.25 (0.04-0.46)

Switzerland	23973.2 (13338.2-35033.4)	279.5 (156.3-406.8)	28429.6 (14772.8-44461.3)	222 (116.8-343.9)	-0.67 (-0.78--0.56)
Syrian Arab Republic	9508.2 (4910.2-14848.2)	141.6 (73.1-218)	20245.9 (10744.9-30998.9)	117.9 (62-181.6)	-0.67 (-0.77--0.58)
Taiwan (Province of China)	22666.2 (12099.2-33465.6)	120.9 (64.4-179.8)	41092.1 (21725.1-62881.6)	117.9 (63.2-179.9)	-0.04 (-0.16-0.09)
Tajikistan	3567.1 (1892.3-5590.5)	111.7 (59.8-172.4)	5367.2 (2758.8-9433.8)	66.9 (34.2-117.9)	-1.61 (-1.69--1.53)
Thailand	35060.2 (20081.6-52928.6)	79 (43.9-120.3)	54768.1 (29060.5-85035)	55.7 (29.5-85.8)	-1.04 (-1.17--0.9)
Timor-Leste	362.3 (199.7-547.1)	82.1 (44.7-127.4)	665.4 (375.8-997)	69.2 (38.8-103.7)	-0.58 (-0.61--0.56)
Togo	1073.1 (534.1-1742.2)	63.5 (31.4-103.5)	2115.8 (1018.1-3830.1)	38.6 (18.6-69.5)	-1.94 (-2.26--1.61)
Tokelau	1.9 (1-3)	146.2 (80.7-230.1)	2.4 (1.4-3.9)	148.8 (85-237.8)	0.08 (0.01-0.14)
Tonga	81.2 (44.2-128.5)	130.7 (70.5-206.5)	90.3 (48.6-141.8)	104 (55.9-163.3)	-0.83 (-0.87--0.78)
Trinidad and Tobago	813.8 (427.6-1344.6)	84.3 (44.3-138.7)	1456.5 (760.7-2515.1)	79.9 (41.4-138.3)	-0.06 (-0.1--0.02)
Tunisia	7295.9 (3954.9-10831.8)	124.9 (67.4-186.9)	15833.3 (8829.6-24399)	113.2 (63.1-174.9)	-0.32 (-0.37--0.28)
Turkey	85652 (48442.7-125960.2)	197.1 (111.2-291.6)	162257 (88804-250014.2)	165.4 (90.8-254)	-0.59 (-0.69--0.5)
Turkmenistan	3130.8 (1696.1-4827.4)	131.8 (72.3-201.1)	5161.4 (2831.8-8534.6)	99 (54.6-163.7)	-1.09 (-1.26--0.92)
Tuvalu	8.3 (4.1-13.2)	105.3 (52.4-167.5)	10.3 (5.6-16.3)	103.2 (57-163.9)	-0.09 (-0.15--0.04)
Uganda	3929.7 (1991.6-6453.1)	49.1 (24.8-80.7)	9721.3 (4889.5-15900.4)	48 (23.1-78.9)	-0.32 (-0.56--0.09)
Ukraine	131218.9 (74566.4-200300)	204.9 (115.8-314.9)	118928.4 (61772.2-179715.5)	192.5 (99.7-291.2)	-0.12 (-0.32-0.07)

United Arab Emirates	1508.2 (734.2-2375.5)	87.3 (41.9-137.4)	9647.7 (4948.9-15932.1)	59.8 (31.1-102.6)	-1.3 (-1.46--1.14)
United Kingdom	181657 (97569.9-269108.6)	258.7 (139.4-376.7)	175153.5 (91330.9-267709.8)	186 (101.9-279.4)	-0.97 (-1.06--0.88)
United Republic of Tanzania	13184.2 (6971.6-20636.6)	97 (51.3-149.8)	31264.4 (16582.2-49844.4)	86 (45.2-136)	-0.5 (-0.57--0.43)
United States of America	784864.6 (418248.2-1151044.3)	275.3 (147.5-401.4)	835605.4 (405943.7-1268694.9)	191.8 (93.5-285.4)	-0.98 (-1.04--0.93)
United States Virgin Islands	64.8 (32.4-108)	61.3 (30.7-100.8)	61.6 (30.5-99.9)	45.2 (22.2-73.5)	-1.1 (-1.28--0.92)
Uruguay	6296.3 (3382.9-9898.8)	188.2 (102.2-297.7)	7817 (4227.9-11964)	189.3 (101.4-289.3)	-0.13 (-0.22--0.04)
Uzbekistan	11596.4 (6156.9-17814.6)	88.9 (46.6-137.8)	29098.5 (15387.3-48327.7)	86.7 (45.4-142.1)	-0.01 (-0.21-0.19)
Vanuatu	80.3 (41-126.6)	92 (47-141.6)	162.8 (83.4-269.8)	69.5 (35.8-115.6)	-1.17 (-1.28--1.07)
Venezuela (Bolivarian Republic of)	13989.6 (7018.7-23571.6)	108.6 (55.2-182.5)	18989.9 (8344.5-33275)	59.5 (26.1-104.1)	-1.96 (-2.08--1.83)
Viet Nam	39902.7 (22827.2-60396.3)	90.3 (51.3-136.3)	87763.3 (46505.1-130394.8)	76.3 (40.5-113.5)	-0.59 (-0.63--0.55)
Yemen	9746.3 (5127.1-15266.4)	141.7 (74.5-217.3)	24670.5 (12110.3-40218.9)	113.4 (55.5-187.4)	-0.92 (-1.01--0.83)
Zambia	2318.2 (1126.6-3648.5)	67.9 (32.6-109.8)	4237.6 (2230.8-6975.7)	46.8 (23.9-77.1)	-1.58 (-1.74--1.42)
Zimbabwe	4983.3 (2588.2-7587.3)	99.1 (51.1-152.1)	6492.2 (3293.7-10148.8)	66.7 (34.4-103.7)	-1.29 (-1.35--1.24)

Note: Data in parentheses represent the 95% uncertainty intervals (UIs). Rates are reported per 100,000 population.

Abbreviations: ASMR, age-standardized mortality rate; ASDR, age-standardized disability-adjusted life years rate; UI, uncertainty interval.

2. Supplementary Figure 1. Sex-specific trends in the proportion and age-specific DALY rates of low back pain caused by smoking, 1990 −2021

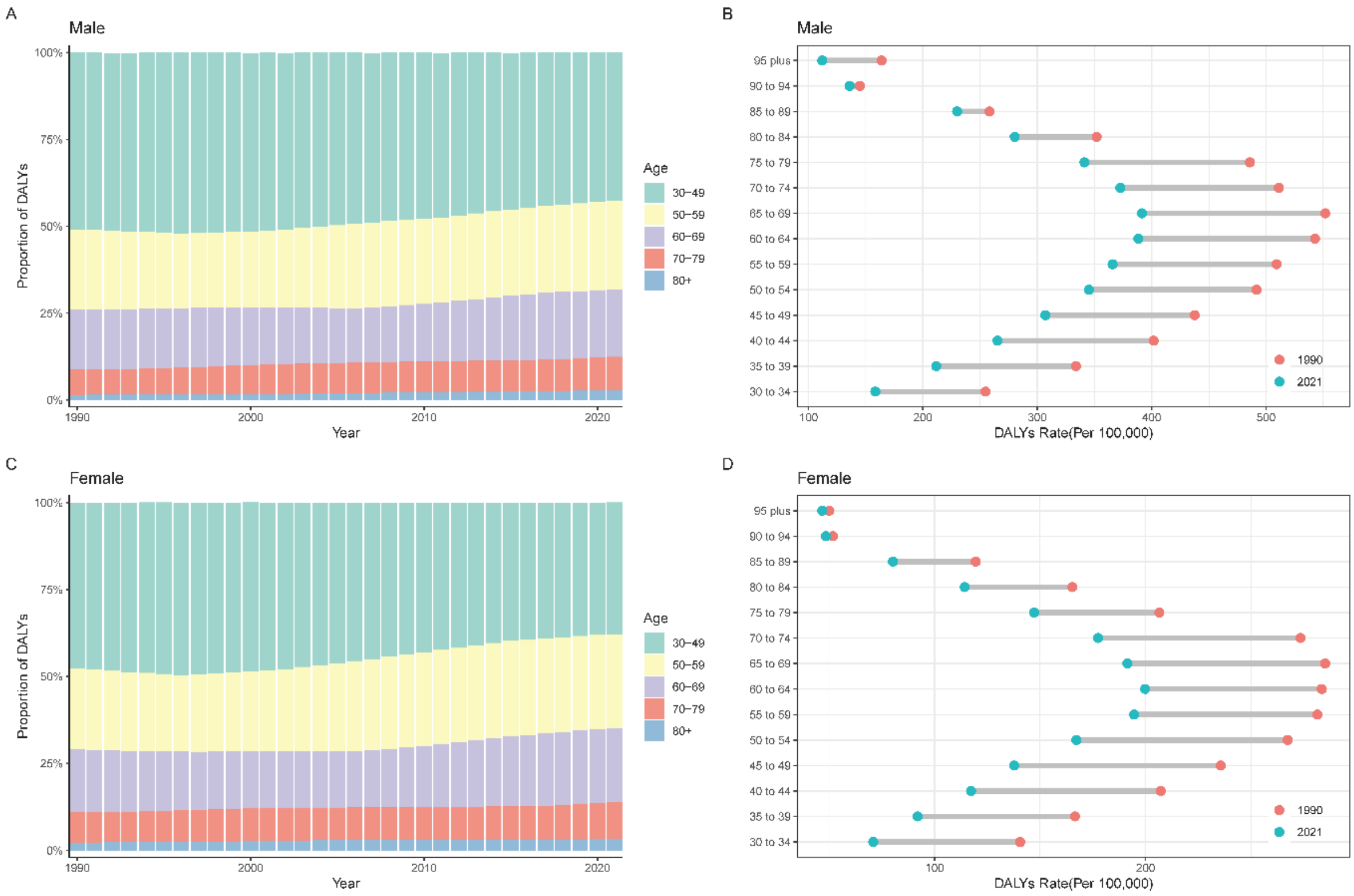

(A) and (C) present the temporal trends in the age composition (proportion) of total DALYs for males and females, respectively, from 1990 to 2021. The distinct colors within the 100% stacked bar charts represent different broad age categories (30-49, 50-59, 60-69, 70-79, and 80+ years). (B) and (D) illustrate the changes in age-specific DALY rates (per 100,000 population) across detailed 5-year age groups for males and females. The dumbbell plots compare the burden between the baseline year 1990 (red dots) and the recent year 2021 (teal dots), with the connecting grey lines indicating the magnitude and direction of the change over the three decades. DALYs: Disability-adjusted life years.

Abbreviations: DALYs, disability-adjusted life years.

=

3. Supplementary Figure 2. Age-period-cohort effects on the DALY rates of low back pain caused by smoking by sex

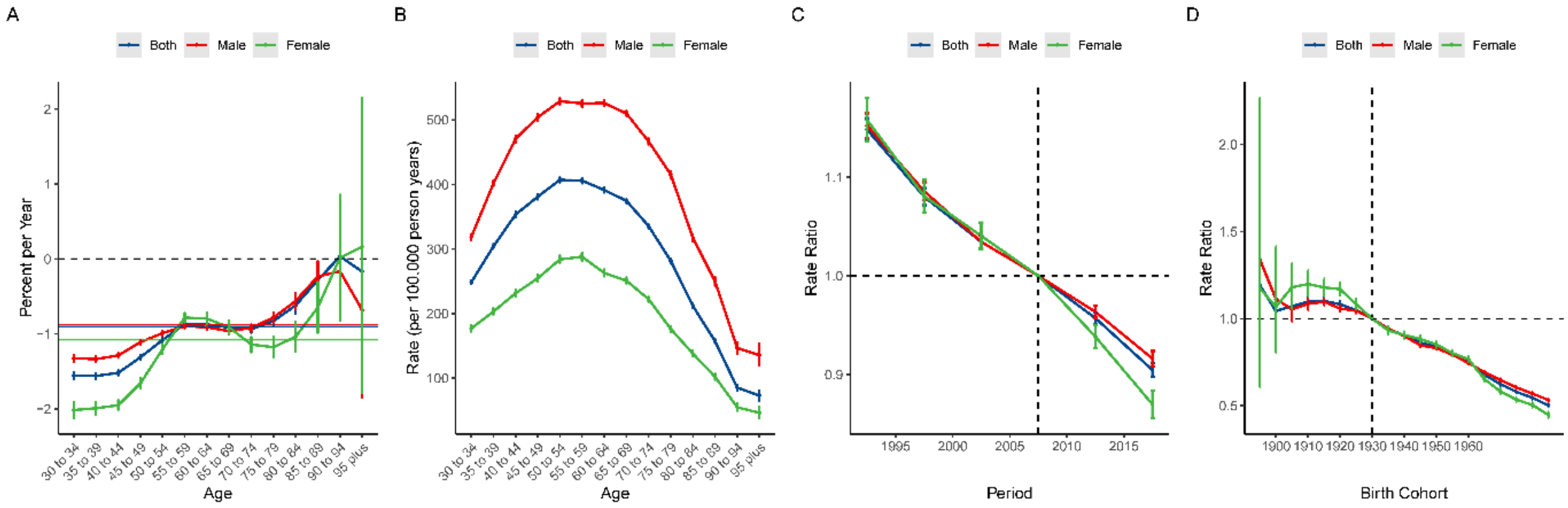

(A) Local drift values, representing the annual percentage changes in the DALY rates across specific age groups. The horizontal dashed line at 0 indicates no percentage change. (B) Longitudinal age curves, indicating the expected age-specific DALY rates (per 100,000 person-years) in the reference cohort, adjusted for period effects. (C) Period relative risks (Rate Ratios) of DALYs adjusted for age and cohort effects. The vertical dashed line indicates the reference period (2007.5), and the horizontal dashed line represents a rate ratio of 1.0. (D) Cohort relative risks (Rate Ratios) of DALYs adjusted for age and period effects. The vertical dashed line indicates the reference birth cohort (1930), and the horizontal dashed line represents a rate ratio of 1.0. In all panels, the blue lines represent both sexes, the red lines represent males, and the green lines represent females. The error bars indicate the 95% confidence intervals (CIs).

Abbreviations: DALYs, disability-adjusted life years; CIs, confidence intervals.

4. Supplementary Figure 3. Joinpoint regression analysis of the age-standardized DALY rates for low back pain caused by smoking globally (Both sexes), 1990 －2021

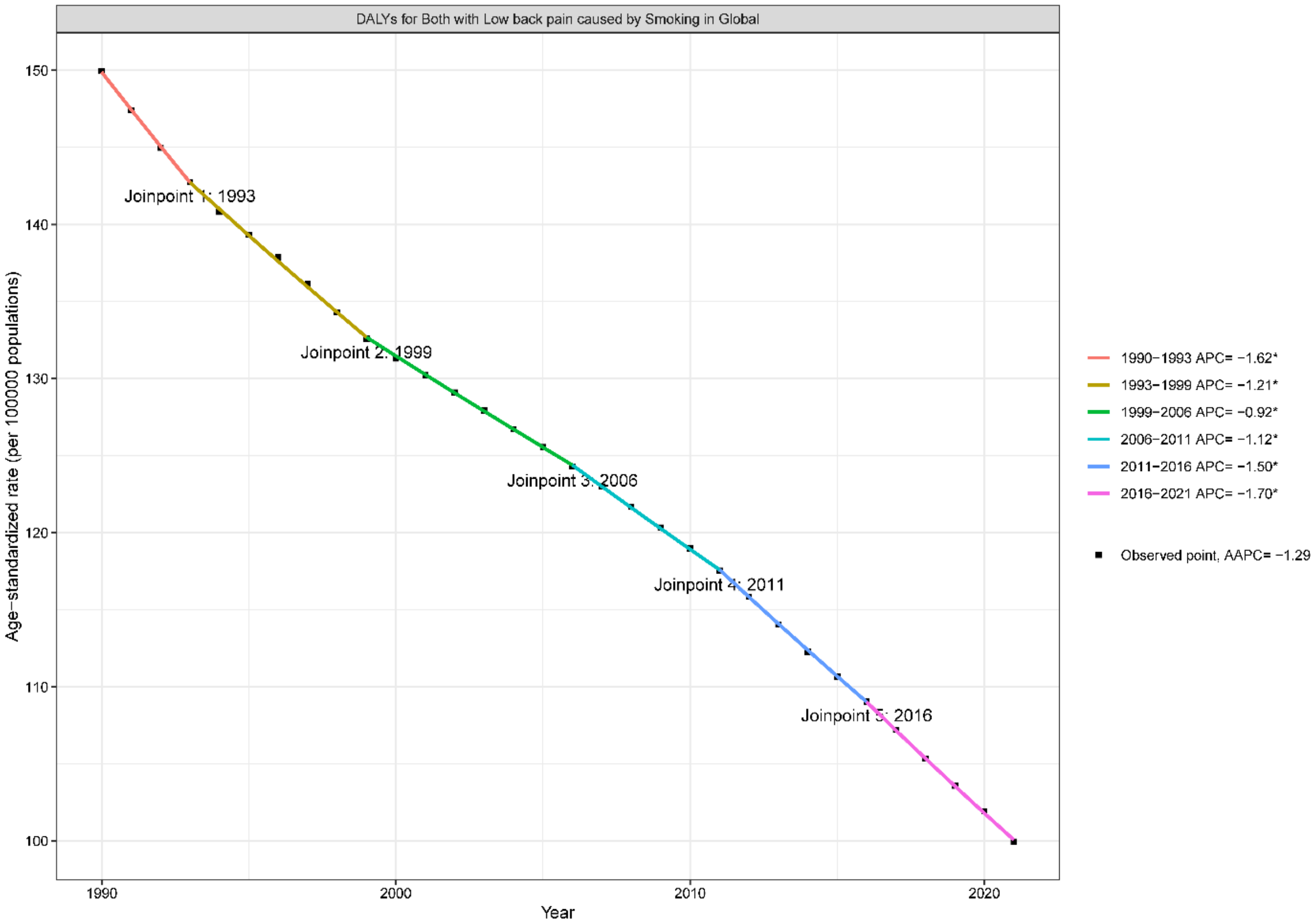

The black square markers denote the observed age-standardized DALY rates (per 100000 population) for each year. The solid colored lines represent the modeled trend segments fitted by the Joinpoint regression analysis. Five distinct joinpoints (inflection points) were identified during the 32-year study period: 1993, 1999, 2006, 2011, and 2016. The embedded legend displays the estimated Annual Percent Change (APC) for each specific trend segment and the overall Average Annual Percent Change (AAPC) for the entire observation period (AAPC = −1.29). An asterisk (*) indicates that the APC is statistically significantly different from zero ($P < 0.05$). Abbreviations: DALYs, disability-adjusted life years; APC, annual percent change; AAPC, average annual percent change.

5. ***Supplementary Figure 4. Joinpoint regression analysis of the age-standardized DALY rates for low back pain caused by smoking globally (Male), 1990 −2021***

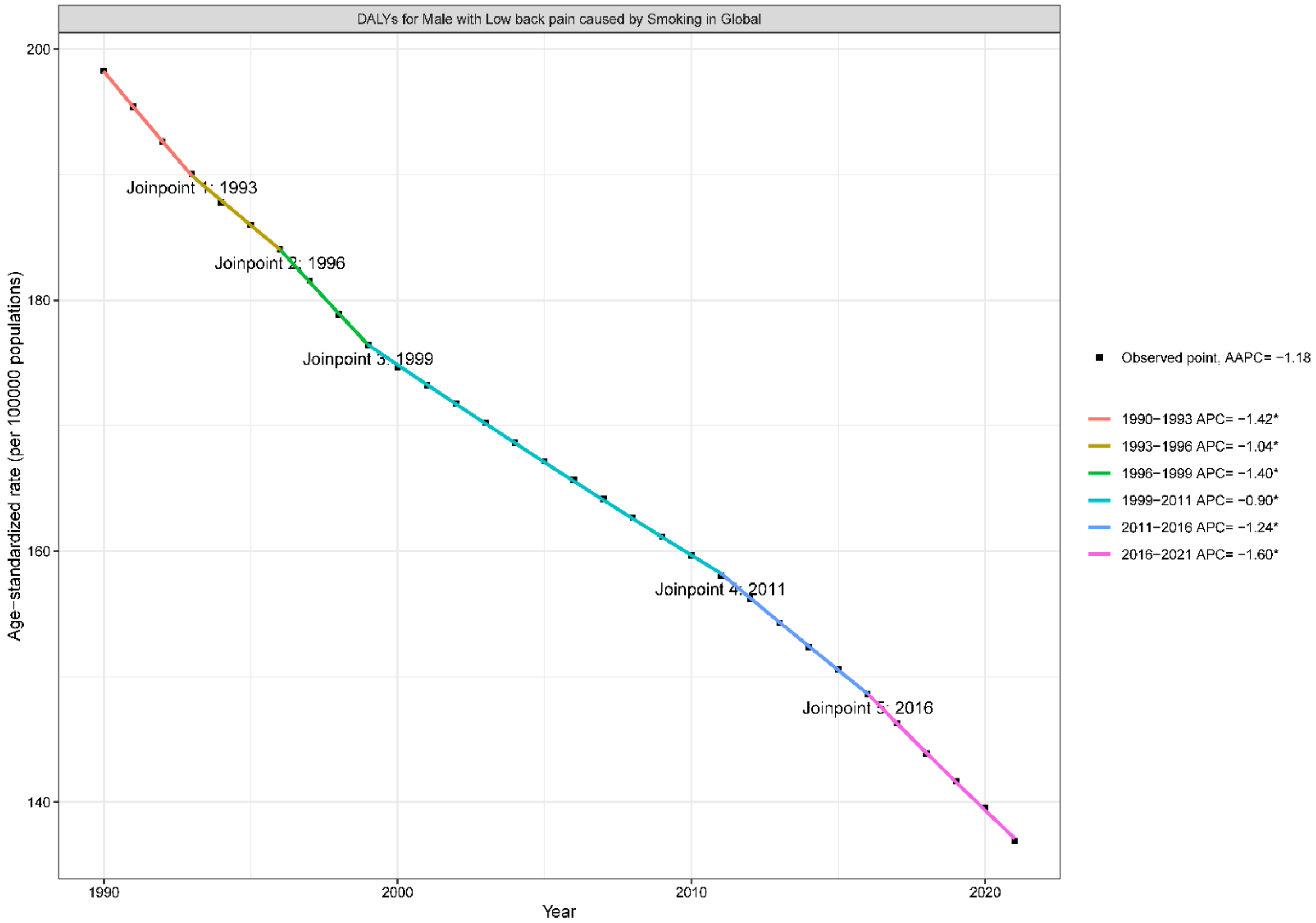

The black square markers denote the observed age-standardized DALY rates (per 100000 population) for males each year. The solid colored lines represent the modeled trend segments fitted by the Joinpoint regression analysis. Five distinct joinpoints (inflection points) were identified during the 32-year study period: 1993, 1996, 1999, 2011, and 2016. The embedded legend displays the estimated Annual Percent Change (APC) for each specific trend segment and the overall Average Annual Percent Change (AAPC) for the entire observation period (AAPC = −1.18). An asterisk (*) indicates that the APC is statistically significantly different from zero ($P < 0.05$).

Abbreviations: DALYs, disability-adjusted life years; APC, annual percent change; AAPC, average annual percent change.

6. Supplementary Figure 5. Joinpoint regression analysis of the age-standardized DALY rates for low back pain caused by smoking globally (Female), 1990 －2021

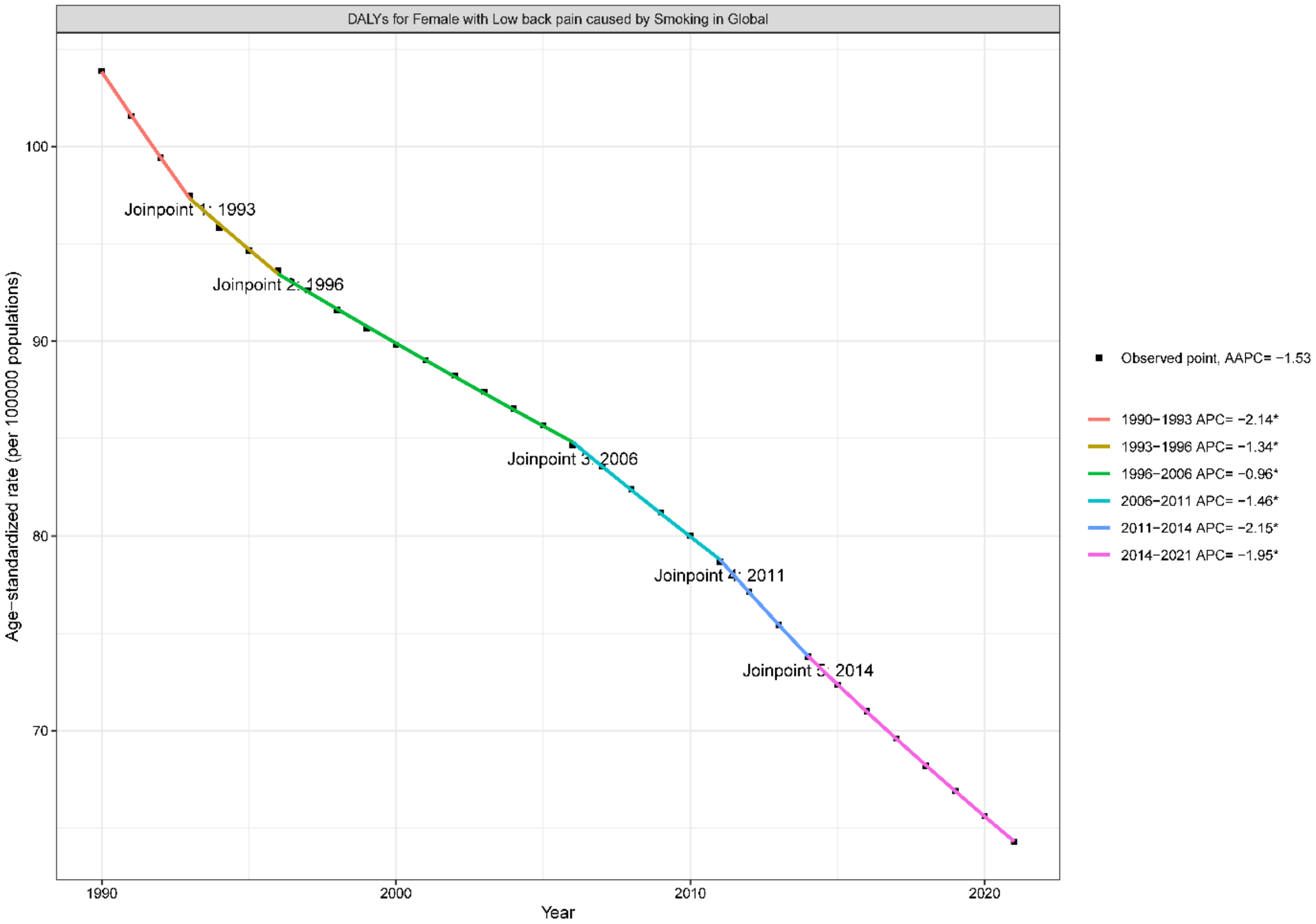

The black square markers denote the observed age-standardized DALY rates (per 100000 population) for females each year. The solid colored lines represent the modeled trend segments fitted by the Joinpoint regression analysis. Five distinct joinpoints (inflection points) were identified during the 32-year study period: 1993, 1996, 2006, 2011, and 2014. The embedded legend displays the estimated Annual Percent Change (APC) for each specific trend segment and the overall Average Annual Percent Change (AAPC) for the entire observation period (AAPC = −1.53). An asterisk (*) indicates that the APC is statistically significantly different from zero ($P < 0.05$).

Abbreviations: DALYs, disability-adjusted life years; APC, annual percent change; AAPC, average annual percent change.
